# Single-cell transcriptome analyses reveal critical regulators of spermatogonial stem cell fate transitions

**DOI:** 10.1186/s12864-024-10072-0

**Published:** 2024-02-03

**Authors:** Shuang Li, Rong-Ge Yan, Xue Gao, Zhen He, Shi-Xin Wu, Yu-Jun Wang, Yi-Wen Zhang, Hai-Ping Tao, Xiao-Na Zhang, Gong-Xue Jia, Qi-En Yang

**Affiliations:** 1 Key Laboratory of Adaptation and Evolution of Plateau Biota, Northwest Institute of Plateau Biology, Chinese Academy of Sciences, Qinghai, 810008 China; 2 University of Chinese Academy of Sciences, Beijing, 100049 China; 3 Qinghai Key Laboratory of Animal Ecological Genomics, Northwest Institute of Plateau Biology, Chinese Academy of Sciences, Qinghai, 810001 China; 4 Institute of Medical Technology, Luoyang Polytechnic, Luoyang, Henan 471000 China

**Keywords:** Spermatogonia, Stem cells, Eomes, Single-cell RNA-seq

## Abstract

**Background:**

Spermatogonial stem cells (SSCs) are the foundation cells for continual spermatogenesis and germline regeneration in mammals. SSC activities reside in the undifferentiated spermatogonial population, and currently, the molecular identities of SSCs and their committed progenitors remain unclear.

**Results:**

We performed single-cell transcriptome analysis on isolated undifferentiated spermatogonia from mice to decipher the molecular signatures of SSC fate transitions. Through comprehensive analysis, we delineated the developmental trajectory and identified candidate transcription factors (TFs) involved in the fate transitions of SSCs and their progenitors in distinct states. Specifically, we characterized the A_single_ spermatogonial subtype marked by the expression of *Eomes*. Eomes^+^ cells contained enriched transplantable SSCs, and more than 90% of the cells remained in the quiescent state. Conditional deletion of *Eomes* in the germline did not impact steady-state spermatogenesis but enhanced SSC regeneration. Forced expression of *Eomes* in spermatogenic cells disrupted spermatogenesis mainly by affecting the cell cycle progression of undifferentiated spermatogonia. After injury, Eomes^+^ cells re-enter the cell cycle and divide to expand the SSC pool. Eomes^+^ cells consisted of 7 different subsets of cells at single-cell resolution, and genes enriched in glycolysis/gluconeogenesis and the PI3/Akt signaling pathway participated in the SSC regeneration process.

**Conclusions:**

In this study, we explored the molecular characteristics and critical regulators of subpopulations of undifferentiated spermatogonia. The findings of the present study described a quiescent SSC subpopulation, Eomes^+^ spermatogonia, and provided a dynamic transcriptional map of SSC fate determination.

**Supplementary Information:**

The online version contains supplementary material available at 10.1186/s12864-024-10072-0.

## Background

Mammalian spermatogenesis is a complex cellular differentiation process that occurs throughout the life of a male. Continual spermatogenesis relies on the function of spermatogonial stem cells (SSCs), which are capable of self-renewal and supplying committed progenitors to sustain or regenerate the whole spermatogenic lineage [1]. SSC activity resides in the undifferentiated spermatogonial population consisting of isolated A_single_ (A_s_), interconnected A_paired_ (A_pr_) and A_aligned_ (A_al_) spermatogonia [2]. Because A_s_ are the most primitive spermatogonia in the adult testis, it was suggested that A_s_ spermatogonia act as SSCs [3]. In contrast, data from lineage tracing experiments indicated that A_pr_ and short chains of A_al_ spermatogonia are not irreversibly differentiated; instead, these cells fragment and revert to become A_s_ spermatogonia and function as stem cells [4]. Both the classic and alternative models agree that a subset of A_s_ spermatogonia are actual stem cells; therefore, identifying the gene expression patterns and regulatory networks that mark this unique cell type is crucial for understanding the molecular regulation of SSC fate decisions.

Heterogeneous gene expression exists within undifferentiated spermatogonia in the mouse testis. While the expression of *Zbtb16*, *Lin28*, *Foxo1*, *Sall4*, *Cdh1* and others distinguishes undifferentiated spermatogonia from the remaining spermatogenic cells [5], some genes are enriched in different subsets of A_s_, A_pr_ or A_al_ spermatogonia. *Id4* was the first marker gene identified in mouse testes that labels A_s_ spermatogonia, which possess potent SSC activities [6]. *Pax7*, *Eomes*, or *Pdx1* is also highly expressed in A_s_ spermatogonia [7, 8]. Notably, the A_s_ spermatogonial population is also not homogeneous and can be further divided into different subfractions based on gene expression and stem cell activities. For example, *Id4* is present in a subpopulation of A_s_ spermatogonia [9]^,^ and *Pax7* marks a very rare subset of A_s_ that are likely negative for *Id4* or *Eomes* [10]. A detailed survey of undifferentiated spermatogonia is needed to further understand the molecular signatures and dynamics of SSCs in the mammalian germline.

Single-cell RNA sequencing (scRNA-seq) is a powerful tool for dissecting the gene expression dynamics and developmental trajectory of stem cells [11]. Data from scRNA-seq analysis of the testis during the fatal and neonatal periods of development provide evidence that SSC fate is predetermined in a subset of prespermatogonia [12]. Recently, the molecular signatures of undifferentiated spermatogonia have been examined, and conserved markers of various spermatogonial subtypes were discovered in different species [13, 14]. However, undifferentiated spermatogonia constitute only 0.3% of germ cells in the testis, and 10% of these cells are A_s_ spermatogonia [15]. Transplantation assays have shown that the testes of adult mice contain approximately 3000 SSCs [16]. Data from single-cell analyses of SSC-containing fractions are still limited. A few studies have examined the gene expression profiles of Id4^+^ and Eomes^+^ cells at the single-cell level [10, 17]; these cells are subpopulations of undifferentiated spermatogonia that possess a proportion of SSC activity. *Eomes*, which is expressed in mesodermal cells, was originally described as a key player in vertebrate embryogenesis [18]. *Eomes* was confirmed to play pivotal roles during anteroposterior axis formation and definitive endoderm specification in mice [19]. *Eomes* is needed for mouse trophoblast development and mesoderm formation, and Eomes loss-of-function mutants are arrested at implantation [20, 21]. However, the function of Eomes in SSC fate decisions is unclear. Furthermore, the involvement of gene regulatory networks in germ cell injury recovery in SSC-containing cells and in subsets of undifferentiated spermatogonia need to be further explored.

In the present study, we aimed to further understand the heterogeneity and characterize the gene regulatory networks of spermatogonia by using scRNA-seq. Importantly, after transplantation, we identified a quiescent Eomes^+^ spermatogonial population that contained SSCs, and their function was assessed during spermatogenesis in mice. These findings provide a comprehensive landscape of transcriptional regulation in the undifferentiated spermatogonial population and reveal a list of genes that may play a role in controlling SSC fate decisions.

## Results

### Single-cell transcriptome landscape of the undifferentiated spermatogonial population

**Fig. 1:**
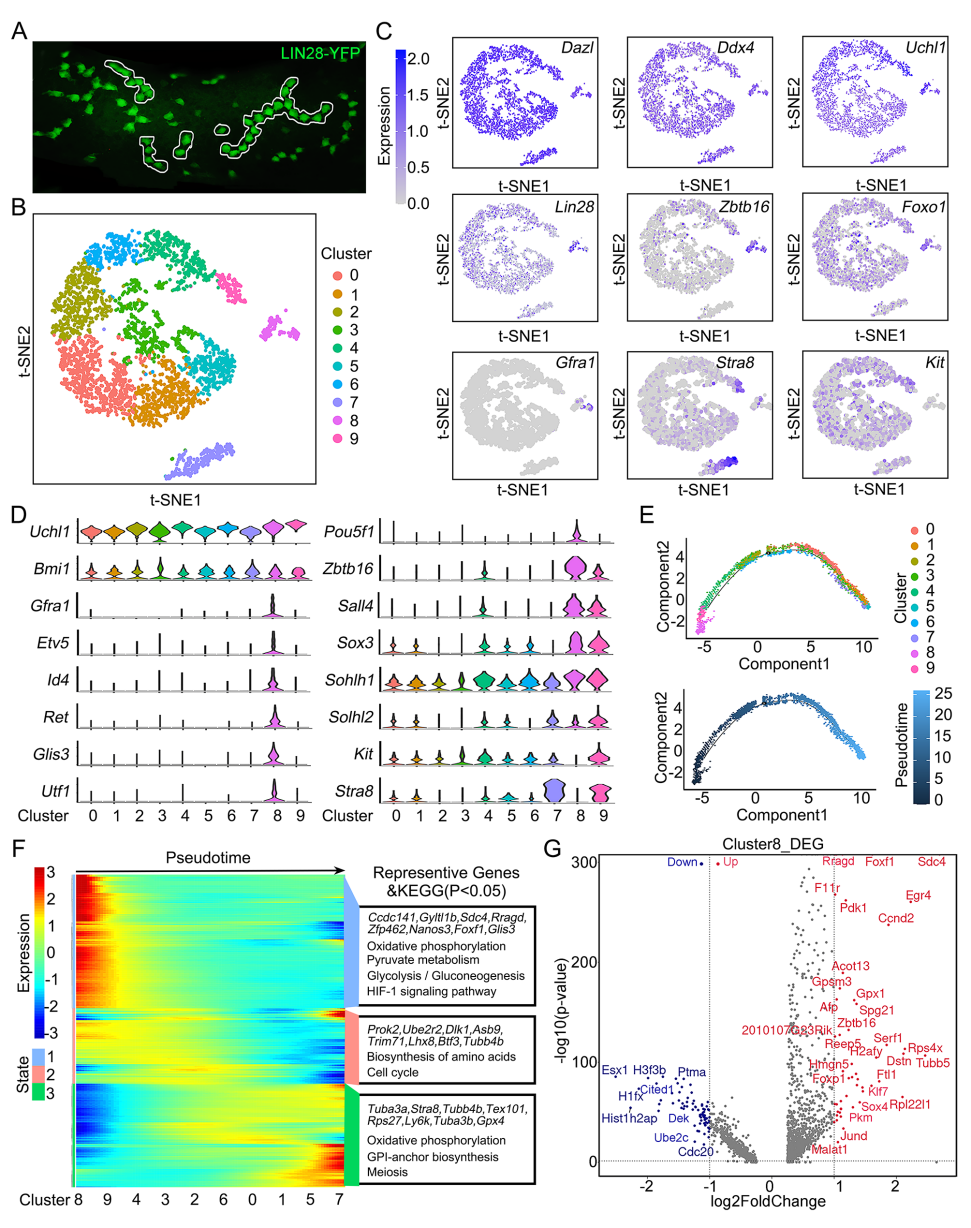
Single-cell RNA-seq analysis of Lin28^+^ spermatogonia isolated from adult testes. **(A)** Whole-mount images of subsets of Lin28-YFP^+^ cells in seminiferous tubules from Lin28-YFP transgenic mice at 2 months. **(B)** t-SNE plot of Lin28-YFP-positive spermatogonial clusters defined by scRNA-seq analysis. Each dot represents a single cell, and clusters are marked by different colors. **(C)** Gene expression patterns of selected marker genes projected on t-SNE plots. **(D)** Violin plots of SSC, progenitor and differentiated spermatogonial marker expression in different clusters. **(E)** Monocle pseudotime trajectory analysis of Lin28-YFP-positive spermatogonial subsets defined in clusters. Cells are marked by pseudotime scores, with dark colors representing immature stages and light colors representing mature stages. **(F)** Heatmaps of pseudotime-dependent genes and enriched KEGG terms for each temporal stage. Blue indicates low expression, and red indicates high expression. **(G)** Volcano plot of genes differentially expressed between putative SSCs in the C8 cluster and other clusters (adjusted *p* value < 0.05; fold change > 2). Red indicates upregulated genes, and blue indicates downregulated genes

Homogenous undifferentiated spermatogonial cells were isolated from the testes of 2-month-old Lin28-yellow fluorescent protein (YFP) knock-in mice [22]. The YFP and endogenous Lin28 signals were colocalized in the same cells, indicating that YFP is a reliable marker for recognizing spermatogonia expressing Lin28 (Fig. S1A). YFP^+^ spermatogonia comprised 3.65 ± 0.60% A_s_, 10.33 ± 0.88% A_pr_ and 86.04 ± 0.56% A_al_ cells (Fig. 1A, S1B). Fluorescence-activated cell sorting (FACS)-isolated YFP^+^ cells were processed for scRNA-seq (*n* = 3 animals) (Fig. S1C- D). A total of 3898 cells passed quality control and were filtered for subsequent analysis. We detected an average of 10,348 copies of transcripts (UMIs) and 4000 genes per spermatogonium (Fig. S1E). The cells were visualized using t-distributed stochastic neighbor embedding (t-SNE), and 10 clusters were identified (Fig. 1B, S1F). Clusters 7 (C7) and 8 were discrete cell types, whereas the remaining clusters followed a continuous order. Preliminary examination of these clusters revealed that all the cells were positive for *Dazl*, *Ddx4*, *Foxo1*, *Zbtb16* and *Uchl1*, indicating no contamination from somatic cells or advanced germ cells (Fig. 1C). The *Lin28*, *Zbtb16*, *Gfra1*, *Foxo1*, *Stra8* and *Kit* transcripts exhibited heterogeneous expression patterns among these clusters, indicating that the Lin28^+^ spermatogonial pool included undifferentiated spermatogonia and a small number of differentiating spermatogonia (Fig. 1C). Evaluation of specific marker genes for these spermatogonial subtypes revealed distinct gene expression dynamics within and across 10 clusters (Fig. 1D). Notably, genes associated with SSC fate (*Id4*, *Gfra1*, *Ret*, *Etv5, Utf1, Glis3*) were highly expressed in C8, while markers of differentiating spermatogonia, including *Stra8* and *c-Kit*, were missing in this cluster. Cells in C8 constituted 4.01% of the Lin28-YFP^+^ cells, which was similar to the percentage of A_s_ spermatogonia revealed by immunofluorescence staining (Fig. S1F). C7 predominantly contained late differentiating spermatogonia that expressed high levels of *Stra8*, *Kit*, *Sohlh1* and *Sohlh2*. Conversely, clusters 0–6 were classified according to the expression of unique gene signatures, and the cells from these clusters exhibited distinct differentiation states (Fig. S1G-H). The cells were likely progenitor spermatogonia that were in different transition phases. The results of transient EdU labeling (2 h) or EdU retention experiments (5 days) revealed that the proliferation of A_s_ spermatogonia was lower, while the EdU retention rate was greater for these cells than for A_pr_ and A_al_ spermatogonia, indicating that the cell cycle status was different in subsets of undifferentiated spermatogonia (Fig. S2A-C). However, analysis of the expression patterns of cell cycle-specific genes showed that most cells in C5 and C6 were in the G2/M phases of the cell cycle, while most cells in C8 and the remaining clusters were in the G0/G1 phase of the cell cycle (Fig. S2D), indicating that the cell cycle status did not dictate clustering.

### Trajectories and molecular features of undifferentiated spermatogonial subtypes

To define the identity and developmental hierarchy of SSCs and their differentiating progenies, trajectory analysis was applied using Monocle 3 (v1.2.7). The outcomes revealed the developmental order of the pseudotime trajectorys with one branching point (Fig. 1E). Specifically, cells from C8 were at the beginning of the trajectory, and cells from C9 were at the early stage of differentiation, followed by C4 and C2 cells. Cells from C5 and C7 were at the end of the trajectory, and other cells were distributed along the pseudotime trajectory (Fig. 1E). Next, we detected changes in gene expression following the undifferentiated spermatogonial trajectory to investigate the cell type signature and candidate genes related to cell fate transitions (Fig. 1F). Overall, 4637 transcripts were differentially expressed, and the SSC-containing C8 population contained the highest number of DEGs among the clusters (*p* value *<* 0.05; Fig. 1G; Supplemental Data 1). By examining the cell type-specific gene expression patterns, we identified genes and pathways that were enriched in SSCs and progenitor spermatogonia at the early, middle, and late stages (Fig. 1F, S3). The SSC-containing population exhibited relatively high expression of *Gfra1*, *Zbtb16*, *Id4*, *Foxo1*, *Glis3*, *Foxc2* and *Utf1*, as did a number of genes that have not been previously linked to SSC fate, including *Ccdc141*, *Gyltl1b*, *Sdc4*, *Rragd*, *Zfp462*, *Foxf1* and others, which are associated with glycolysis and pyruvate metabolism. Cells in the early differentiating state expressed *Prok2*, *Ube2r2*, *Dlk1*, *Asb9* and *Trim71*, and the genes associated mainly with DNA replication and cell cycle control, while those in the late differentiating state expressed higher levels of *Tuba3a*, *Stra8*, *Tex101*, *Rps27*, *Ly6k*, *Tuba3b*, *Gpx4* and genes that mainly participate in meiosis (Fig. S3; supplemental data 2). Together, these analyses revealed the developmental trajectory and gene expression signatures related to the fate transitions of undifferentiated spermatogonia.

### Analysis of transcriptional networks regulating SSC and progenitor states

Because transcription factors (TFs) play central roles in directing cell-specific gene expression and lineage determination [23], a transcriptional network analysis was conducted to screen candidate transcription regulators with cluster-specific expression patterns. We particularly focused on the SSC-containing cluster and detected 1191 upregulated genes and 1710 downregulated transcripts (Fig. 1G; supplemental data 3). The GO terms associated with the upregulated genes were associated with the regulation of transcription, the cell cycle, cell division, translation, stem cell population maintenance and cell migration, while the downregulated genes were associated with the negative regulation of gene expression, the apoptotic process, cell adhesion, translation, covalent chromatin modification and other processes (Supplemental Data 4). Among these genes, 1005 were transcription factors that bind to and potentially direct the expression of 10,057 targets (Supplemental Data 5).

**Fig. 2:**
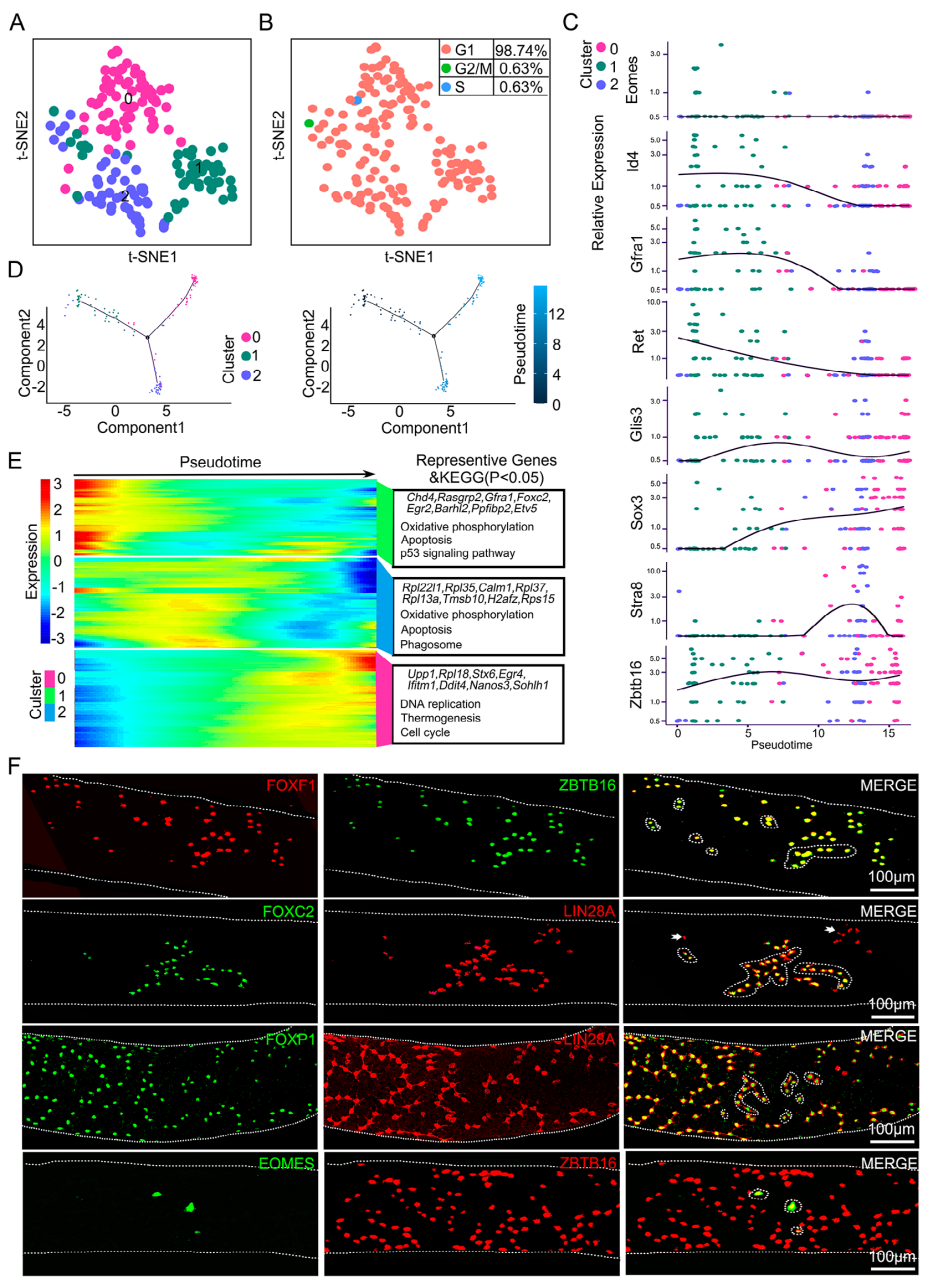
Molecular signatures of SSCs containing undifferentiated spermatogonial subpopulations. **(A)** t-SNE plot of the reclustering of putative SSCs containing spermatogonia. Each dot represents a single cell, and cell clusters are distinguished by color. **(B)** t-SNE plot with cell cycle analysis of putative spermatogonial stem cells. **(C)** Relative expression of markers for SSCs, progenitors and differentiating spermatogonia. **(D)** Pseudotime trajectory analysis of the origin subsets of undifferentiated spermatogonia defined in clusters. Cells are colored according to the pseudotime score, with dark colors representing immature stages and light colors representing mature stages. **(E)** Heatmaps of pseudotime-dependent genes and enriched KEGG terms for each temporal stage. Blue indicates low expression, and red indicates high expression. **(F)** Detection of FOXF1, FOXC2, FOXP1 and EOMES with undifferentiated spermatogonia markers (LIN28A and ZBTB16) by immunofluorescent whole mount staining. Examples of A_s_, A_pr_, and A_al_ (up to 16 interconnected cells) spermatogonia are shown by white dotted lines. White arrows indicate negative expression. Scale bars = 100 μm, *n* = 3

Because C8 cells were not a homogenous fraction, we further separated this population using t-SNE and examined the gene expression profiles of the resulting 3 clusters (Fig. 2A). A major proportion (98.74%) of these cells were in the G1 phase of the cell cycle (Fig. 2B). Gene expression and developmental trajectory analysis revealed that the cells in C1 were the most primitive cellular fraction (Fig. 2C, D). Transcripts associated with SSC activities were enriched in C1 cells (*Chd4, Rasgrp2, Egr2*, *Gfra1*, *Foxc2*, and *Barhl2*), and genes related to progenitor fate were upregulated in C0 and C2 cells (*Calm1, Nanos3*, *Ddit4*, *Upp1*, *Stx6*, *Egr4* and *Sohohl1*) (Fig. 2E). The upregulated genes in the C1 cluster were associated with small GTPase-mediated signal transduction and stem cell population maintenance, while the downregulated genes were involved mainly in proliferation (Fig. 2E). This cluster also showed enrichment of more than 68 TFs (Fig. S5; supplemental data 5), including *Foxm1*, *Tcf3*, *Mesp1*, *Foxc2*, *Sox2*, *Zfp42*, *Zfp143*, *Msx2* and *Klf7* (*p* value < 0.01) (Fig. S5). *Foxm1* participates in spermatogonial regeneration after busulfan-induced testicular injury [8]. *Foxc2* appears to have a functional role in sustaining SSCs in primary cultures of spermatogonia [24]. Visualization analysis of the transcriptional networks revealed candidate TFs, including *Eomes*, *Id4*, *Foxc2*, *Zic1*, *T*, *Zbtb16*, *Foxo1*, *Foxp1*, *Hoxc4*, and *Rest, via Cytoscape (v3.8.2)* (Fig. S4A-B). These transcripts were enriched in SSCs and progenitor spermatogonia and may play important roles in regulating SSC fate decisions (Fig. S4A-B). Next, we selected *Eomes*, *Foxf1*, *Foxc2* and *Foxp1* to test whether these TFs were present in undifferentiated spermatogonia at the protein level using immunohistochemical or whole mount staining of seminiferous tubules. The results confirmed that the majority of FOXC2 and FOXP1 proteins were colocalized with ZBTB16 or LIN28A (Fig. 2F, S4C-D). FOXF1 was not expressed in some A_s_ or short chains of A_al_ spermatogonia, while an immunoreactive signal for EOMES was observed only in A_s_ and A_pr_ spermatogonia (Fig. 2F, S4C-D). Collectively, the outcomes of these single-cell transcriptomic analyses provided a unique and potentially invaluable database for discovering important factors directing the fate decisions of different spermatogonial subpopulations.

### Identification and characterization of a novel SSC population labeled by *Eomes* expression

**Fig. 3:**
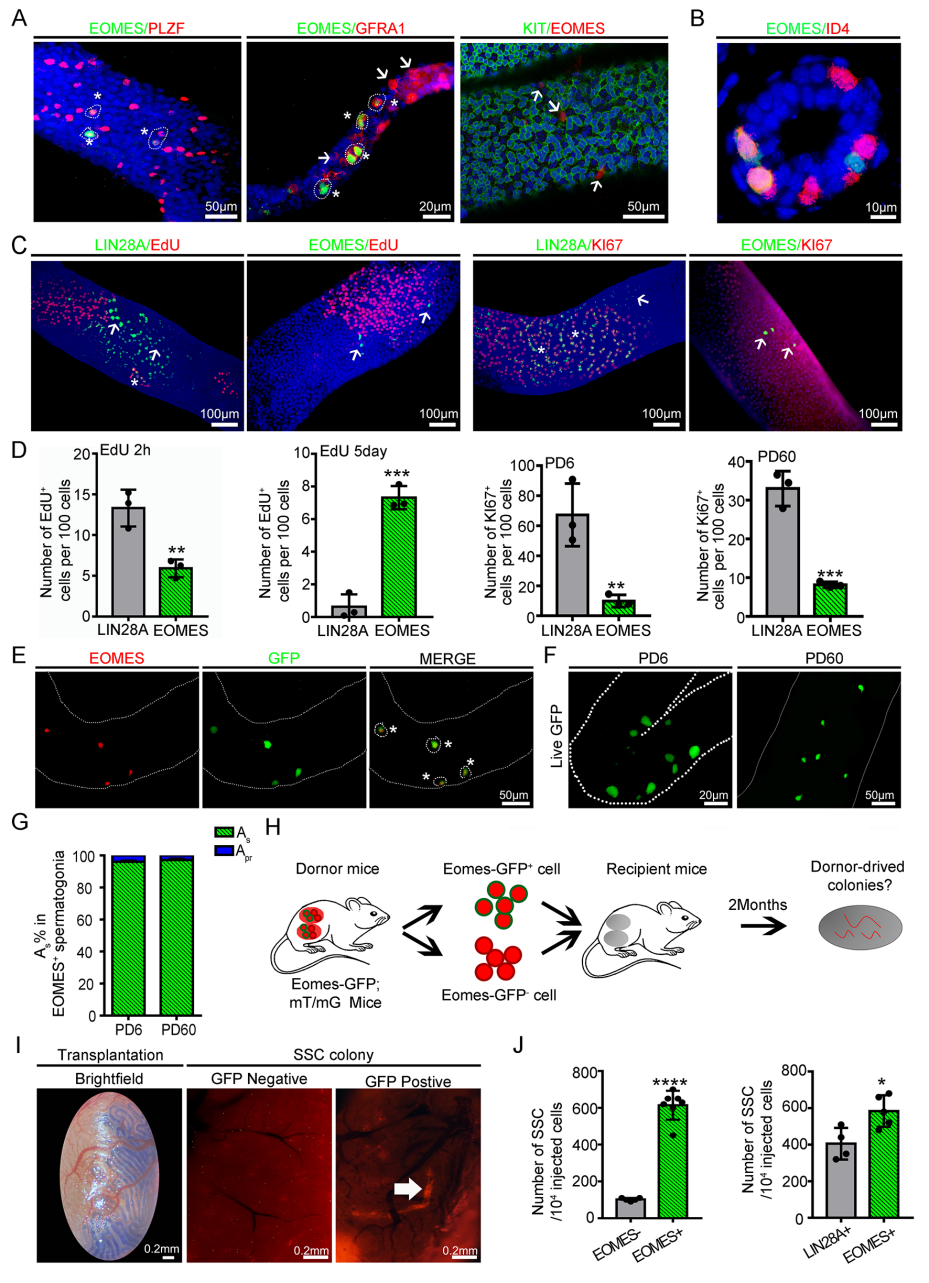
Eomes-positive spermatogonia are a subpopulation of undifferentiated spermatogonia that function as SSCs. **(A)** Whole-mount immunostaining of PLZF (ZBTB16) (PD60), GFRA1 (PD6) and c-KIT (PD6) with EOMES in seminiferous tubules. Scale bars = 20 μm/50 µm, *n* = 3. **(B)** Coimmunostaining of EOMES and ID4 in testes from 6-day-old mice. Scale bar = 10 μm, *n* = 3. **(C)** Whole-mount immunostaining of EdU and LIN28A, Ki67 and LIN28A, EdU and EOMES, and Ki67 and EOMES in seminiferous tubules 2 h after EdU injection. Scale bars = 100 μm, *n* = 3. **(D)** Quantification of EdU^+^ and ^Ki67+^ spermatogonia in Lin28A^+^ cells and EOMES^+^ cells of testes at PD6 and PD60. At least 1500 LIN28A^+^ cells and 500 EOMES^+^ cells were counted at each time point. *n* = 3. *p value < 0.05, **p value < 0.01, and ***p value < 0.001. **(E)** Whole-mount immunostaining of EOMES and GFP expression in seminiferous tubules from Eomes-GFP transgenic mice at PD60. Scale bar = 50 μm, *n* = 3. **(F)** Whole-mount image of live seminiferous tubules from Eomes-GFP transgenic mice at PD6 and PD60. Scale bars = 20 μm/50 µm, *n* = 6. **(G)** Percentages of A_s_ or A_pr_ spermatogonia in Eomes^+^ cells. At least 500 EOMES^+^ cells were counted at each time point. *n* = 6. (H) Schematic diagram of the SSC transplantation experiment. **(I)** Microscopic examination of testes after receiving Eomes^+^ cells and Eomes^−^ cells. Scale bars = 0.2 mm, *n* = 6. **(J)** Donor-derived spermatogenic clones of Eomes^+^, Lin28^+^ cells and Eomes^−^ cells. SSC numbers were derived from donor-derived colonies of spermatogenesis in recipient testes and normalized to the 10^4^ cells injected. * *p* value < 0.05 and **** *p* value < 0.0001. Each dot represents a biological repetition

Although the abovementioned analyses identified a list of potentially important transcription regulators in Lin28^+^ spermatogonia, the number of A_s_ was still limited, thus hindering the attempt to comprehensively understand this spermatogonial population. To address this limitation, we next focused on Eomes^+^ spermatogonia, which consist primarily of A_s_ cells. Eomes^+^ cells were a fraction of undifferentiated spermatogonia (Zbtb16^+^, Gfra1^+^ and cKit^−^) and were primarily present as A_s_ and A_pr_ spermatogonia (Fig. 3A). Importantly, a proportion of the Eomes^+^ spermatogonia lacked debatable Id4 expression at both the mRNA and protein levels (Id4^+^Eomes^−^, 59.16 ± 4.22%; Id4^−^Eomes^+^, 6.67 ± 1.85%; and Id4^+^ Eomes^+^, 34.17 ± 4.85%) (Fig. 3B). Results of EdU incorporation assays confirmed that Eomes^+^ spermatogonia were slow-cycling cells because only 5.92% of these cells were positive for EdU staining, in contrast to 46.8% of Lin28^+^ cells in the adult testis (Fig. 3C- D). Similarly, the results of whole-mount immunostaining of KI67 and LIN28A and Ki67 and EOMES in seminiferous tubules confirmed that Eomes^+^ spermatogonia were slow-cycling cells because only 8.16% of these cells were positive for Ki67 staining, in contrast to 33.02% of Lin28^+^ cells in the adult testis (Fig. 3C-D). In addition, 5 days after EdU^+^ injection, 10.22% of the Eomes^+^ cells retained the EdU signal, which was significantly greater than the 0.39% of the Lin28^+^ cells or 2.9% of the A_s_ spermatogonia (Fig. 3C- D, S6A-C). This feature was already established in the foundational undifferentiated spermatogonial pool at PD6 (Fig. S6A-C).

Moreover, 90.17% of the Eomes cells were negative for the proliferative index Ki67 (PD6), suggesting that these cells were quiescent (Fig. 3D). Next, we used the transplantation assay, the gold standard of the SSC test, to evaluate whether Eomes^+^ cells exhibited SSC activity. To this end, an Eomes-GFP/RFP double transgenic line was generated to isolate Eomes^+^ and track the fate of cells after transplantation. All GFP cells were stained by endogenous *Eomes* in the seminiferous tubules; more than 96% of the Eomes-GFP^+^ cells were A_s_ spermatogonia, and less than 4% of these cells were A_pr_ cells, indicating that the transgene faithfully marked the Eomes^+^ cells (Fig. 3E-G). FACS-isolated Eomes^+^ spermatogonia were transplanted into the testes of busulfan-treated recipients, and stem cell-derived colonies were quantified (Fig. 3H). The results showed that SSC activity was strongly enriched in Eomes^+^ cells compared to in Eomes-depleted testicular cells (614 ± 29.83 vs. 102 ± 3.74 colonies per 10^5^ cells transplanted) (*p* value < 0.05, *n* = 7) (Fig. 3I - J). Histological analyses revealed that donor-derived spermatogenic cells and sperm were present in the recipient testis 2 months after transplantation (Fig. S6D). There were more SSCs generated from Eomes^+^ cells than from Lin28^+^ cells (584 ± 38.68 vs. 405 ± 43.3 colonies per 10^5^ cells transplanted) (*p* value < 0.05, *n* = 5) (Fig. 3J), confirming that the Eomes^+^ cells contained abundant SSCs. The gene expression patterns of *Eomes* projected on the t-SNE plots in C8 of Lin28-YFP single cells showed that the Eomes^+^ cells were a subpopulation of Lin28^+^ cells (Fig. S7A). Lin28-YFP^+^ spermatogonia and Eomes-GFP^+^ spermatogonia were integrated (Fig. S7B-D), and cell cycle analysis suggested that Eomes^+^ spermatogonia contained more quiescent cells than Lin28^+^ cells (Fig. S7E). Moreover, *Eomes* was detected in spermatogonia from the seminiferous tubules of humans, monkeys, cattle, and cats (Fig. S8), indicating that Eomes is a conserved marker of primitive spermatogonia in mammalian testes.

### Functional roles of *Eomes* in regulating SSC fate decisions revealed by loss- and gain-of-function experiments

**Fig. 4:**
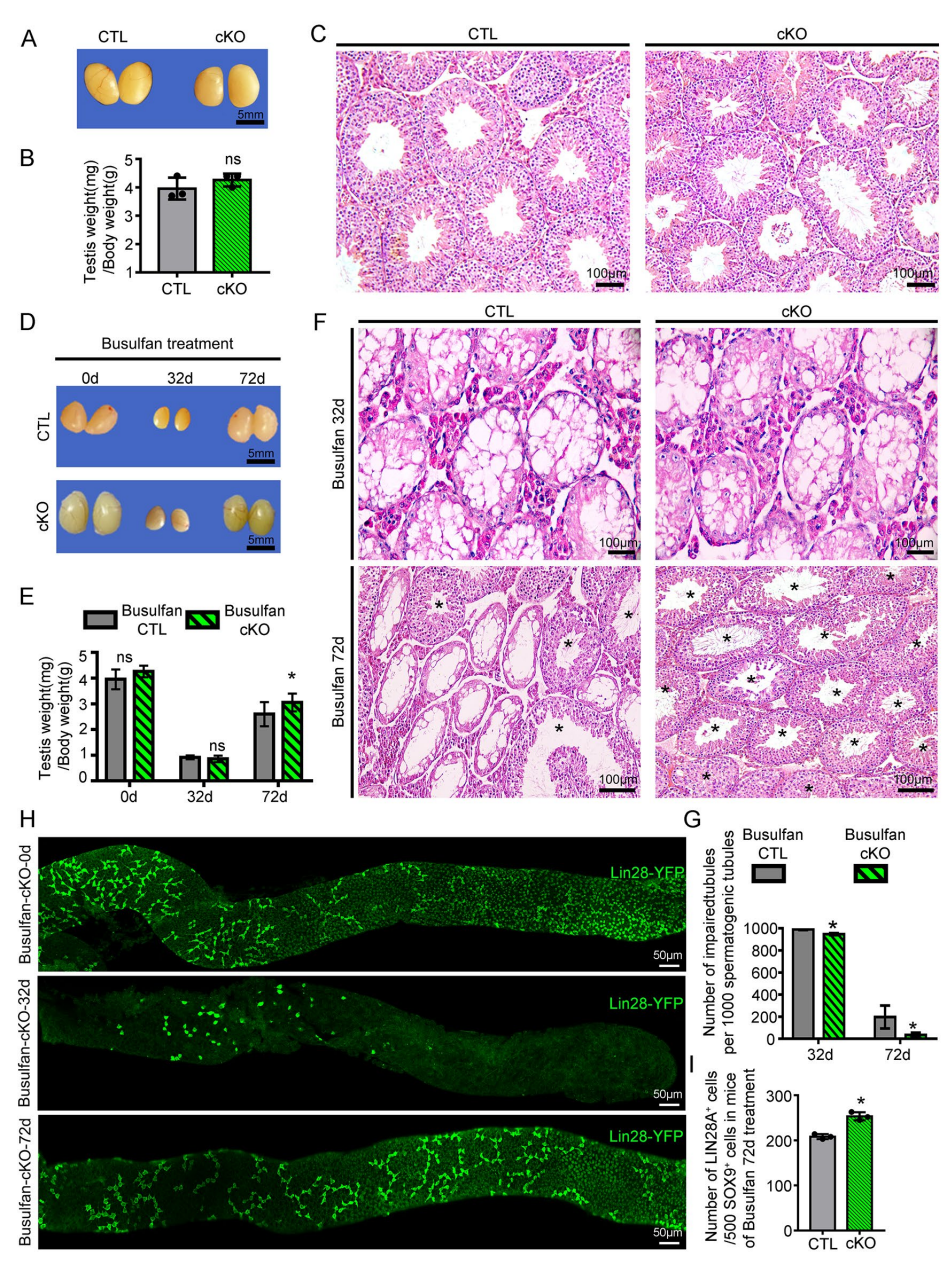
Eomes deletion enhanced SSC regeneration after busulfan-induced testicular injury. **(A)** Representative images of testes from control and Eomes-cKO mice at PD90. Scale bar = 5 mm, *n* = 3. **(B)** Telephone (mg)-to-body weight (g) ratios of control and Eomes-cKO mice; ns, not significant; *n* = 3. **(C)** Hematoxylin and eosin (H&E)-stained testicular cross-sections from control and Eomes-cKO mice at PD90. Scale bars = 100 μm, *n* = 3. **(D)** Representative images of testes from control and Eomes-cKO mice at 0, 32 or 72 days postbusulfan treatment. Scale bar = 5 mm, *n* = 5. **(E)** Testes (mg)-to-body weight (g) ratios of control and Eomes-cKO mice at 0, 32 or 72 days postbusulfan treatment. *p value < 0.05, ns means not significant, *n* = 5. **(F)** Representative images of H&E-stained testicular cross-sections of control and Eomes-cKO mice at 32 or 72 days postbusulfan treatment. Asterisks indicate seminiferous tubules with complete recovery of spermatogenesis. Scale bars = 100 μm, *n* = 5. **(G)** Quantification of damaged seminiferous tubules in the testes of control and Eomes-cKO mice 32 or 72 days after busulfan treatment. A total of 1000 spermatogenic tubules were quantified. *n* = 5, *p value < 0.05. **(H)** Representative images of whole-mount immunostaining of LIN28A^+^ spermatogonia in the seminiferous tubules of busulfan-treated testes at different time points. Scale bars = 50 μm, *n* = 3. **(I)** Quantification of LIN28A^+^ spermatogonia per 500 Sertoli cells in testicular cross-sections of control and Eomes-cKO mice at 72 days postbusulfan treatment. At least 500 Sox9^+^ cells were counted for each sample. *n* = 5, *p value < 0.05

These data and a previously published lineage tracing experiment support the statement that Eomes^+^ cells perform unique functions in SSC homeostasis [10]; unfortunately, whether *Eomes* play any functional roles in spermatogenesis remains unclear. To address this knowledge gap, an *Eomes* conditional knockout mouse model (Eomes-cKO) was generated by crossing *Eomes*^*flox/flox*^ animals with *Ddx4-Cre mice*. *Eome* ablation (Fig. S9A) did not cause major defects in spermatogenesis because testicular weight did not differ between control and Eomes-cKO animals (Fig. 4A- B). Histological examination revealed complete spermatogenesis in control and Eomes-cKO animals (Fig. 4C). The total numbers of spermatogenic cells and undifferentiated spermatogonia per 500 Sertoli cells were comparable between control and Eomes-cKO mice (Fig. S9B-C). To investigate the impact of *Eomes* loss-of-function on SSC behavior during generation, we treated Eomes-cKO animals with 20 mg/kg busulfan and investigated the dynamics of spermatogenic recovery. Interestingly, 32 days after busulfan treatment, the testis weights were similar between the control and Eomes-cKO animals, and 72 days after treatment, the testis weights increased (75.80 ± 7.34 mg vs. 62.40 ± 7.46 mg control), and the number of recovered seminiferous tubules in the knockout mice was significantly greater than that in the control mice (965.70 ± 12.77 vs. 802.80 ± 46.44) (Fig. 4D-G). Similarly, the relative number of undifferentiated spermatogonia marked by LIN28A was increased in Eomes-cKO animals at the end of recovery (Fig. 4H-I). These findings revealed a novel role of *Eomes* as a negative regulator of SSC function, and its deletion enhanced spermatogonial regeneration after injury.

**Fig. 5:**
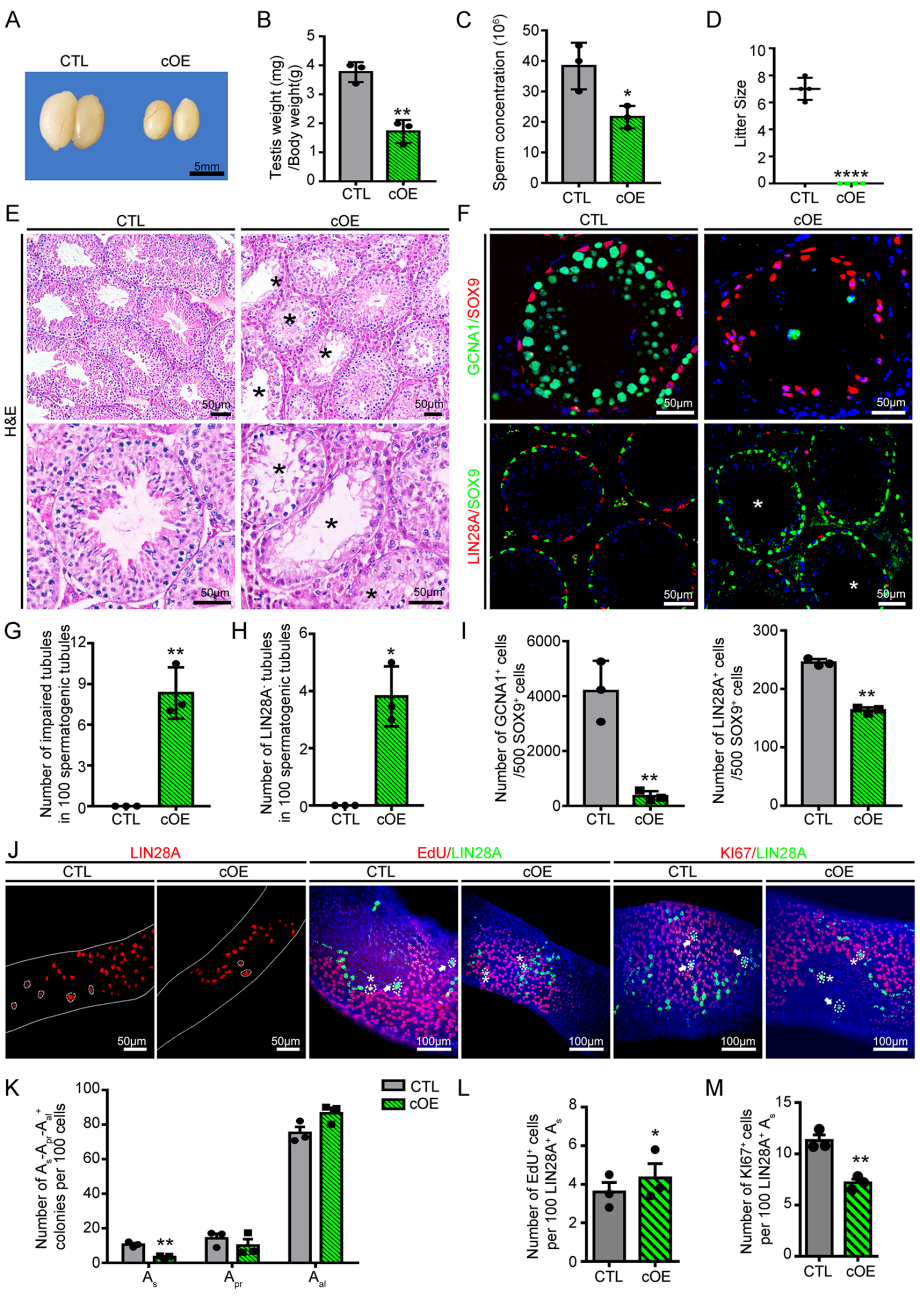
Forced expression of Eomes in the germline impairs spermatogenesis and disrupts the homeostasis of undifferentiated spermatogonia. **(A)** Representative image of testes from control and *Eomes* conditional overexpression (Eomes-cOE) animals at PD90. Scale bar = 5 mm, *n* = 3. **(B)** Ratios of testes (mg) to body weight (g) of control and Eomes-cOE mice at PD90; **p value < 0.01, *n* = 3. **(C)** Sperm concentrations of control and Eomes-cOE mice at PD90; *p value < 0.05, *n* = 3. **(D)** Fertility test of control and Eomes-cOE mice after pairing with wild-type females; ****p value < 0.0001, *n* = 3. **(E)** H&E-stained testicular cross-sections of control and Eomes-cOE mice. The black asterisks indicate seminiferous tubules with impaired spermatogenesis. Scale bars = 100 μm, *n* = 3. **(F)** Immunofluorescence staining of testicular cross-sections from control and Eomes-cOE mice. Germ cells, Sertoli cells and undifferentiated spermatogonia were stained with GCNA1, SOX9, and LIN28A, respectively. White asterisks indicate seminiferous tubules devoid of undifferentiated spermatogonia. Scale bars = 50 μm, *n* = 3. **(G)** Quantitative analyses of seminiferous tubules with impaired spermatogenesis in control and Eomes-cOE animals. A total of 1000 spermatogenic tubules were quantified; **p value < 0.01, *n* = 3. **(H)** Quantification of seminiferous tubules devoid of undifferentiated spermatogonia in control and Eomes-cOE animals. A total of 1000 spermatogenic tubules were quantified; *p value < 0.05, *n* = 3. **(I)** The number of germ cells and undifferentiated spermatogonia per 500 Sertoli cells in testicular cross-sections of control and Eomes-cOE animals. At least 500 Sox9 + cells were counted for each sample; **p value < 0.01, *n* = 3. **(J)** Whole-mount immunostaining of LIN28A, Ki67 and EdU in the seminiferous tubules of control and Eomes-cOE mice. Spermatogonia are shown by white dotted lines. Scale bars = 50 μm/100 µm, *n* = 3. **(K)** Quantitative comparisons of the proportions of A_s_, A_pr_ and A_al_ spermatogonial cohorts in control and Eomes-cOE mice. At least 1500 LIN28A + cells were counted for each sample; **p value < 0.01, *n* = 3. **(L)** Percentage of A_s_ spermatogonia among LIN28A^+^ cells colocalized with EdU and **(M)** Ki67 from control and Eomes-cOE mice. The data are presented as the mean ± s.e.m. for at least 3 independent experiments. At least 1500 LIN28A + cells were counted for each sample; *p value < 0.05 and **p value < 0.01

Based on these findings, we questioned whether forced expression of *Eomes* could change SSC fate decisions and result in defects in spermatogenesis. To explore this possibility, a transgenic mouse model was generated in which Eomes were conditionally overexpressed in the germline using *Dddx4-cre* (Eomes-cOE). A transgene was assembled with a flox-stop sequence inserted between the constitutive human ubiquitin C (UBC) promoter and *Eomes* coding sequence (Fig. S10A), and the activation of Cre removed flox-stop sites to induce *Eomes* expression only in germ cells. Compared with those in age-matched littermate controls, eomes transcript levels were increased by 10.42-fold (Fig. S10B-D), and the testes/body-weight ratio was greatly reduced in the eomes-cOE males (1.72 ± 0.23 vs. 3.78 ± 0.19 control, *p* value < 0.05; *n* = 3, *p* value *<* 0.05) (Fig. 5A- B). The Eomes-cOE males were completely sterile due to the low sperm concentration despite the normal sperm morphology (Fig. 5C- D, S10E). The histology of the seminiferous tubules of testes from Eomes-cOE males was severely disrupted (Fig. 5E), and the number of germ cells was significantly reduced (Fig. 5F-I). The number of germ cells in PD0 testes did not differ between control and Eomes-cOE animals, indicating that Eomes overexpression in fetal testes did not cause detectable abnormalities in germ cells (Fig. S11 A- B); however, the number of LIN28A^+^ cells began to decrease at PD8 (Fig. S11 C- D). Seminiferous tubules devoid of undifferentiated spermatogonia appeared at 3 months of age, confirming the impact of forced *Eomes* expression on the SSC pool (Fig. 5F-I). Among the different subsets of undifferentiated spermatogonia, the number of A_s_ cells was significantly decreased by *Eomes* overexpression (3.37 ± 0.62 vs. 7.86 ± 1.15 control) (Fig. 5J-K). The reduction in A_s_ spermatogonia was likely due to increased apoptosis and elevated proliferation. The percentage of TUNEL and LIN28A double-positive cells was increased by 59.63% (6.96 ± 0.63% vs. 4.36 ± 0.37% control) in the seminiferous tubules of Eomes-cOE animals (Fig. S11 E-F). EdU incorporation into A_s_ spermatogonia increased by 20.28% (4.33% ± 0.74 vs. 3.60% ± 0.50 control), and the number of Ki67^+^ cells decreased by 37.84% (8.16% ± 0.43 vs. 33.02% ± 2.61 control) in the seminiferous tubules of Eomes-cOE animals (Fig. 5L- M, S10F), further demonstrating that *Eomes* overexpression changed cell cycle dynamics. In summary, these data supported the conclusion that forced *Eomes* expression in the germline results in defects in spermatogonial fate decisions.

### scRNA-seq analysis revealed distinct molecular and cellular features of Eomes^+^ spermatogonia after germ cell injury

**Fig. 6:**
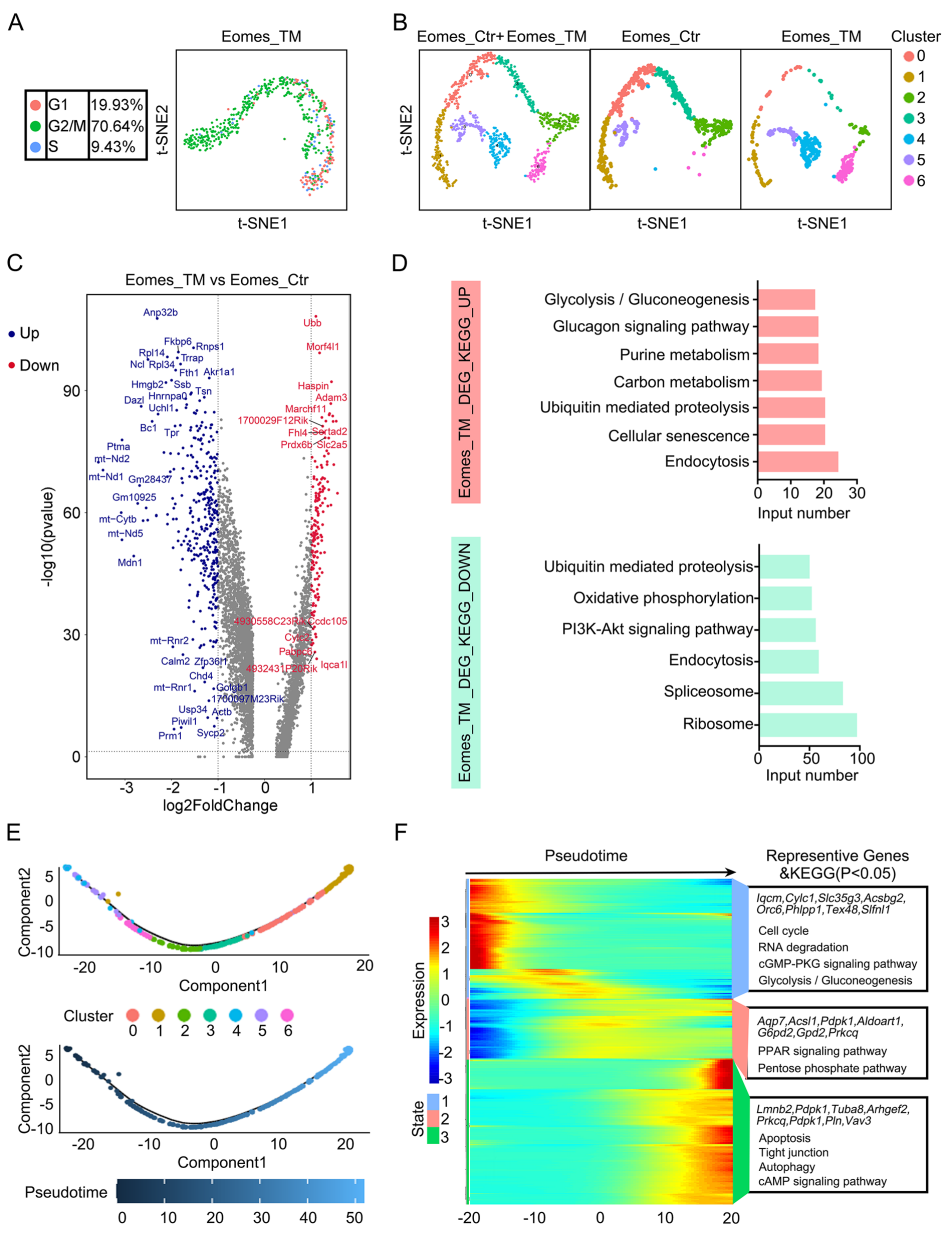
**(A)** Molecular signatures of Eomes^+^ germ cells from mice after busulfan treatment for 8 days. Cell cycle analysis of Eomes^+^ germ cells from Eomes-TMs was performed using Seurat, reputation = t-SNE. The G1, S and G2/M phases are indicated by the corresponding colors. **(B)** t-SNE plot of integrated Eomes + spermatogonia from Eomes-Ctr and Eomes-TM. Each dot represents a single cell, and cell clusters are distinguished by color. **(C)** Volcano plot of genes differentially expressed between the Eomes-TM and Eomes-Ctr groups (adjusted *p* value < 0.05; fold change > 2). Red indicates upregulated genes, and blue indicates downregulated genes. **(D)** Enriched KEGG terms for DEGs in Eomes + spermatogonia from the Eomes-TM cohort. **(E)** Pseudotime trajectory analysis of integrated Eomes + spermatogonial subsets defined in clusters. Cluster 4 was the developmental origin subpopulation. Cells are colored according to the pseudotime score, with dark colors representing immature stages and light colors representing mature stages. **(F)** Heatmap of 3 distinct states of pseudotime-dependent genes and enriched KEGG terms for each temporal state. Blue indicates low expression, and red indicates high expression

We were interested in the molecular mechanism by which *Eomes*, as a negative regulator of SSC function, enhances spermatogonial regeneration after injury, and a germ cell injury mouse model was developed. After low-dose busulfan (20 mg/kg body weight)-induced testis injury, on day 8, the numbers of EdU^+^ and Ki67^+^ cells increased by 3.8- and 4.4-fold, respectively (Fig. S12). We conducted scRNA-seq analyses on FACS-isolated Eomes-GFP^+^ cells from the testes of 8-week-old mice (Eomes-Ctr) and low-dose busulfan (20 mg/kg body weight, day 8)-induced testis injury mice (Eomes-TM). A total of 4820 cells and 8679 cells passed quality control, and the mean numbers of transcripts with unique molecular identifiers were 6069 and 5425, respectively (Fig. S13A-B). Unsupervised clustering via t-SNE analysis revealed that the cells were distributed into 4 or 5 different clusters (Fig. S13C-D). Cell cycle analysis confirmed that Eomes-GFP^+^ cells from the testes of Eomes-TMs re-entered the cell cycle because 19.93% of these cells were in G0/G1 phase, 9.43% were in S phase and 70.64% were in G2/M phase (Fig. 6A). These data showed that the cell cycle status of Eomes^+^ cells was dramatically different between steady-state spermatogenesis and spermatogenic injury/regeneration.

Next, we integrated the Eomes-Ctr and Eomes-TM datasets to identify TF regulators that may play important roles in regulating the undifferentiated state of the spermatogonial lineage in damaged germ cells. Evaluation of specific marker genes for these spermatogonial subtypes revealed distinct gene expression dynamics within and across 7 clusters (Fig. 6B, S13E). Notably, genes associated with SSC fates (*Id4* and *Gfra1*) were highly expressed in C4, C5 and C6, while markers of differentiating spermatogonia, including *Stra8* and *c-Kit*, were missing in all the other clusters (S12E). Cluster 4 and cluster 6 were distinctly more abundant in Eomes-TMs than in Eomes-Ctr (Fig. 6B). We first examined the differences in the gene expression of Eomes-GFP^+^ cells in the Eomes-TM group compared to the Eomes-Ctr group. A total of 5036 genes were differentially expressed (Fig. 6C, Supplemental Data 6). The upregulated genes were associated with glycolysis/gluconeogenesis, the glucagon signaling pathway, and purine metabolism, and the downregulated genes were associated with the mTOR signaling pathway, the pI3k-Akt signaling pathway, and oxidative phosphorylation (Fig. 6D, Supplemental Data 6).

Pseudotime trajectory analysis revealed that cluster 4 was the initial population, followed by cluster 5, cluster 6, cluster 2, cluster 3, cluster 0 and cluster 1 in continuous order (Fig. 6E). Next, we detected changes in gene expression following the Eomes^+^ spermatogonial trajectory to investigate the cell type signature and candidate genes related to cell fate transitions (Fig. 6F). By examining the cell type-specific gene expression patterns, we identified genes and pathways that were enriched in Eomes^+^ spermatogonia after germ cell injury (Fig. 6F). *Iqcm*, *Cylc1*, *Slc35g3*, *Acsbg2* and *Orc6* cells and genes associated mainly with the cell cycle and glycolysis/gluconeogenesis were expressed in the SSC state, while those in the differentiating state expressed higher levels of *Aqp7*, *Acsl1*, *Pdk1*, *Aldoart1* and *Gpd2* and genes that mainly participate in the pentose phosphate pathway. Furthermore, a subpopulation of cells expressed *Lmnb2*, *Pdpk1*, *Tuba8*, *Arhgef2*, and *Prkcq*, as well as genes that are mainly associated with apoptosis and autophagy.

## Discussion

It has been known for decades that undifferentiated spermatogonia are a heterogeneous germ cell population that contains stem spermatogonia and their transit-amplifying progenies [25]. However, the cellular and molecular features of these compartments have remained largely unclear until recently. A previous study collected approximately 12,000 cells from the adult mouse testis [26]^,^ and another study used 897 Eomes^+^ cells for scRNA-seq analyses [10]. In this study, we conducted single-cell analysis of isolated Lin28^+^ spermatogonia, and a total of 3898 Lin28^+^ spermatogonia and 13,499 Eomes^+^ spermatogonia were collected for scRNA-seq. Through comprehensive analysis, we revealed the gene expression signatures of different spermatogonial subsets. We also identified candidate regulators of SSC fate decisions, particularly those related to the stem cell content and gene expression of a quiescent SSC population marked by *Eomes* in the germline.

The undifferentiated spermatogonial population consists of subsets of germ cells with different transcription and chromatin accessibility signatures. It was suggested that undifferentiated spermatogonial subpopulations are transcriptionally plastic and lack distinct molecular states [26]. RNA velocity analysis of Id4-GFP spermatogonia revealed distinct gene expression patterns and cell cycle distributions within SSCs and progenitors [14]. We investigated the cellular heterogeneity of Lin28^+^ spermatogonia and examined the transcriptomes of 10 different undifferentiated spermatogonial subsets. Through integrated analysis, we dissected changes in transcription associated with cell fate transitions and identified a list of transcription regulators that likely play crucial roles in SSC maintenance, progenitor expansion and spermatogonial differentiation. For instance, the forkhead box (FOX) family transcription factors *Foxf1* and *Foxp1* are specific to undifferentiated spermatogonia in the germline. *Foxf1* directs hematopoietic lineage commitment during embryogenesis [27]. *Foxp1* regulates cardiac morphogenesis during the midgestational period of development [28]. High-throughput functional screening will be useful for elucidating the roles of these factors in undifferentiated spermatogonial development and spermatogenesis.

One of the important findings is that *Eomes* expression labels a quiescent SSC population. Undifferentiated spermatogonia in stages II-VII of the seminiferous tubules exhibit low proliferative activity and are largely quiescent [29]. Recent studies have provided important evidence that a quiescent subpopulation of undifferentiated spermatogonia acts as primitive SSCs and that the transition to the active state requires mTOR activity [14]. It appears that DNA methylation also has an important role in maintaining SSC quiescence [30]. Deletion of Dnmt3l disrupts the balance between cycling and the quiescent state within the undifferentiated spermatogonial compartment [31]. In humans, quiescent SSCs are self-renewing cells that function as the ultimate germline stem cells in the testis [32]. It was proposed that both ultimate and transitory SSCs exist in mice and that the two distinct states are interconvertible [33]. *Id4* is expressed in a small fraction of spermatogonia, and cells expressing high levels of this transcription inhibitor have been reported to be the ultimate SSCs [9]. Whether Id4^+^ SSCs are in a quiescent or proliferative state has not been determined, and it appears that some A_pr_ and A_al_ spermatogonia also stain for ID4 [10]. The transcription factor *Eomes* is expressed in a subpopulation of slow-cycling spermatogonia, and a majority of Eomes^+^ cells are negative for *Id4*, raising the possibility that this subset of spermatogonia indeed serves the functions of ultimate or primitive SSCs. The data from our study indicated that a subset of Eomes^+^ cells are quiescent SSCs with unique transcriptome signatures. Like other types of quiescent stem cells [34], Eomes^+^ spermatogonia are characterized by a lack of *Ki67* expression, low thymidine analog incorporation, and unique gene expression patterns. Genes regulating the hypoxia response, pyruvate metabolism and oxidative phosphorylation were differentially expressed within distinct Eomes^+^ subpopulations. RNA velocity analysis revealed that quiescent Eomes^+^ cells were in a transient state, indicating that these cells were arrested during the cell cycle and did not reversibly exit the cell cycle. Serial transplantation or long-term lineage tracing is needed to determine the cell cycle dynamics of these cells.

Eomes^+^ spermatogonia likely act as a reservoir of SSCs and play crucial roles in regeneration. After busulfan-induced testicular injury, the cell cycle and developmental trajectory of Gfra1^+^ spermatogonia change dramatically, and specifically, quiescent SSCs are activated to enhance regenerative capacity [8]. In agreement with previous findings, our analysis revealed that Eomes^+^ cells are resistant to busulfan treatment and proliferate after insult, suggesting that these cells are regenerative SSCs. Eomes^+^ cells are a heterogeneous population that includes 7 different subtypes, and how these cells sense regenerative signals and initiate the cell cycle to promote SSC recovery is poorly understood. Quiescent neural stem cells can be reactivated by insulin from the niche via PI3/Akt signaling [35]. Quiescent long-term hematopoietic stem cells enter the cell cycle after infection or chemotherapy to replenish the whole blood lineage after receiving signals from vascular niches [36]. SSCs reside in a vasculature-associated niche environment [37], and it will be interesting to dissect the niche components of Eomes^+^ cells within the seminiferous tubules because a subset of these cells expresses a greater number of genes related to the hypoxia response.

Another novel finding is that *Eomes* performs important functions as a potent regulator of SSC fate decisions. *Eomes* participates in the first lineage segregation during early embryonic development, and its deletion results in failure of trophoblast stem cell development [38]. *Eomes* and its closely related transcription factor *Brachyury* (*T*) simultaneously repress pluripotency and promote germ-layer formation by changing the chromatin landscape [39]. *Eomes* can act as a transcription activator or repressor to control the expression of specific genes, therefore dictating the lineage decisions of stem cells [40]. One action of *Eomes* is to inhibit retinoic acid (RA) signaling, and the antagonization of RA-induced differentiation provides a crucial mechanism for preserving stem cells [41]. Eomes^+^ spermatogonia do not express c-KIT, which is a marker of differentiating spermatogonia and is induced by RA in the germline [42]. We speculated that the presence of *Eomes* in spermatogonia prevents RA-induced differentiation. Genetic ablation of *Eomes* did not change steady-state spermatogenesis; however, it increased the regenerative capacity of SSCs. A recent study revealed that deletion of *Tgr5* enhances spermatogonial recovery after exposure to busulfan [43]. This phenotype mimics what we observed in Eomes-cKO animals. In sharp contrast, forced expression of *Eomes* decreased the number of SSCs by changing the cell cycle dynamics of undifferentiated spermatogonia. Whether *Eomes* function is compensated by *T* in spermatogonia remains to be tested, although transcriptional network analysis indicated that these two factors are functionally connected in undifferentiated spermatogonia. The outcomes of an ongoing study in which a *T* and *Eomes* conditional double knockout animal model was used will provide a definitive answer.

## Conclusions

In this study, we generated a single-cell gene expression atlas of purified undifferentiated spermatogonia containing SSCs and progenitor spermatogonia isolated from adult murine testes. We then conducted an integrated analysis to uncover a list of transcription regulators that may play a role in regulating SSC fate decisions. Finally, we identified a slow-cycling SSC population marked by the expression of Eomes and provided solid evidence that Eomes plays a functional role in SSC regeneration and cell cycle regulation. We also described the transcriptome of Eomes^+^ spermatogonia during regeneration and provided a dynamic transcriptional map of SSC fate determination.

## Materials and methods

### Animals

All animal experiments were performed in accordance with the Guide for the Care and Use of Laboratory Animals and were approved by the Animal Welfare and Ethics Committee at the Northwest Institute of Plateau Biology, Chinese Academy of Sciences. The Lin28-YFP knock-in [22], *Eomes*^*flox/flox*^ [44], and RFP (Jackson Laboratories, stock no. 002073) mouse lines were maintained on the C57BL/6J background. The *Eomes-GFP* transgenic line [45] and Ddx4-cre [46] were maintained on the FVB background. Eomes^fl/fl^ females were mated with *Ddx4-cre* males to generate *Ddx4-cre; Eomes*^*fl/+*^ males. Young *Ddx4-cre*; *Eomes*^*fl/+*^ males (< 12 weeks old) were crossed with *Eomes*^*fl/fl*^ females to generate Eomes-cKO and *Ddx4-cre; Eomes*^*+/−*^ (littermate control) mice. *Eomes* conditional overexpression animals were generated by using zygote microinjection (Supplemental Materials). For genotyping, genomic DNA was extracted from tail tips and assayed using polymerase chain reaction (PCR) primer sets for alleles (Supplemental Table 1).

All operations were carried out on 8–10-week-old male mice that were intraperitoneally injected with a mixture of busulfan (Cat. No. B2635; Sigma‒Aldrich) (low dose: 20 mg/kg body weight; for SSC transplantation: 44 mg/kg). Intraperitoneal injection of busulfan was performed as described by Morimoto et al. [47].. Briefly, busulfan was dissolved in dimethyl sulfoxide (DMSO; Cat. No. D8418; Sigma Chemical) at a concentration of 20 mg/mL. Just before the injection, an equal volume of heated (37 °C) sterile distilled water was added to reach a final concentration of 10.0 mg/mL. Notably, the solubility of busulfan is poor, and busulfan/DMSO was mixed with water just before use to prevent precipitation.

### Single-cell RNA-Seq

Lin28-YFP^+^ cells and Eomes-GFP^+^ cells were isolated using FACS, and scRNA-Seq libraries were generated using the 10X Genomics Chromium Controller Instrument and Chromium Single Cell 3’ V2 Reagent Kits (10X Genomics, Pleasanton, CA) as described previously [48, 49]. Briefly, cells were concentrated to 1000 cells/µL, and approximately 10,000 cells were loaded into each channel to generate single-cell Gel Bead-In-Emulsions (GEMs), which resulted in the expected mRNA barcoding of 6,000 single cells for each sample. The amplified barcoded cDNA was fragmented, A-tailed, ligated with adaptors and index PCR amplified. The final libraries were quantified using the Qubit High Sensitivity DNA Assay (Thermo Fisher Scientific), and the size distribution of the libraries was determined using a high sensitivity DNA chip on a Bioanalyzer 2200 (Agilent). All the libraries were sequenced on a HiSeq Xten platform (Illumina, San Diego, CA) via a 150 bp paired-end run.

We applied fastq with default parameter filtering of the adaptor sequence and removed the low-quality reads to obtain clean data. Then, the feature barcode matrices were obtained by aligning the reads to the mm10 genome using CellRanger v2.0.0. Cells containing “2500 > nFeature_RNA > 200” expressed genes and a percentage of mitochondrial genes < 25 were removed from the expression table but used for cell expression regression to avoid the effect of the cell status on the clustering analysis and marker analysis of each cluster. The Seurat package (version: 3.2.0, https://satijalab.org/seurat/) was used for cell normalization and regression based on the expression table according to the UMI count of each sample and percentage of mitochondria to obtain the scaled data. PCA was performed based on the scaled data with all highly variable genes, and the top 10 principals were used for t-distributed stochastic neighbor embedding (t-SNE) construction. Utilizing the graph-based clustering method, we acquired unsupervised cell cluster results based on the top 10 principal components of PCA, and we calculated the marker genes by the FindAllMarkers function with the Wilcoxon rank sum test algorithm under the following criteria: (1) logFC > 0.25; (2) *p* value < 0.05; and (3) min.pct > 0.1. We applied single-cell trajectory analysis utilizing Monocle3 (http://cole-trapnell-lab.github.io/monocle-release) using DDR-Tree and default parameters. Before Monocle analysis, we selected marker genes from the Seurat clustering result and raw expression counts of the cells that passed filtering.

### Transcription factor regulon prediction

Assessment of transcription factors was performed using the R package SCENIC (version 1.2.4). To run the SCENIC workflow on our scRNA-Seq data, the GENIE3 (v1.12.0) input matrix was used to infer transcription factors and candidate target genes based on coexpression. The indirect targets were pruned from these modules using the cis-regulatory motif discovery (cisTarget, v1.10.0) algorithm with default parameters. To predict transcription factor regulons, we used the mouse version 9 motif collection, as well as both the mm9-500 bp-upstream-7species.mc9nr.feather and mm9-tss-centered-10 kb-7species.mc9nr.feather databases from cisTarget (https://resources.aertslab.org/cistarget/). The activity of these regulons was quantified via an enrichment score for the regulon target genes (AUCell, v1.12.0). The resulting AUC scores per cell and adjacency matrix were used for downstream analysis and visualization. The heatmap shows all regulons in random cells in the cell matrix. Each row in the figure represents a regulon, each column is a cell, and the color represents the AUC value.

### Histological and immunofluorescence staining

Testicular tissues were fixed in Bouin’s solution for histological analysis via hematoxylin and eosin staining. Testicular tissues were fixed in 4% paraformaldehyde (PFA) for immunofluorescence staining of cross-sections or whole mount seminiferous tubules as described previously [50]. After antigen retrieval in 10 mM sodium citrate (pH 6.0), the slides were washed and then blocked for 1 h in 10% donkey serum (Solarbio, SL050). The sections were incubated with primary antibody at 4 °C overnight and then incubated with the secondary antibody for 2 h. Specimens were mounted for observation under a Nikon fluorescence microscope (Nikon, ECLIPSE E200, Japan) equipped with a CCD camera (Tusen, China).

### SSC transplantation

Eomes^+^ or Lin28^+^ cells isolated from male RG mice (mT/mG mice) were transplanted into the seminiferous tubules of busulfan-treated recipient mice as described. Briefly, FACS-isolated cells were resuspended in mouse serum-free medium [9] at 1 × 10^6^ cells/mL, and 10 µL (10,000 cells) was microinjected into each recipient testis. Recipient testes were evaluated for colonies of donor-derived spermatogenesis 2 months later.

### Statistical analysis

There were more than three samples collected in each group. Experimental data were collected, and all values are expressed as the means ± standard errors. The data were analyzed with GraphPad Prism software version 7.00 (www.graphpad.com). Differences between means were examined using t tests and one-way analysis of variance (ANOVA) followed by Tukey’s test. A *p* value < 0.05 indicated statistical significance.

### Electronic supplementary material

Below is the link to the electronic supplementary material.

Supplementary Material 1

Supplementary Material 2

Supplementary Material 3

Supplementary Material 4

Supplementary Material 5

Supplementary Material 6

Supplementary Material 7

Supplementary Material 8

Supplementary Material 9

## Data Availability

The authors declare that the data supporting the findings of this study are available within the paper and its supplementary materials or are available from the corresponding author upon reasonable request. Single-cell RNA-seq datasets have been uploaded to NCBI (BioProject: PRJNA981678 and PRJNA758213).
