# Supplementary material for: Single-cell transcriptome analyses reveal critical regulators of spermatogonial stem cell fate transitions
Source: BMC Genomics. 2024 Feb 3;25:138. doi: 10.1186/s12864-024-10072-0 (PMC10837949; doi:10.1186/s12864-024-10072-0)
Supplement: Supplementary file 1 — Supplementary Material 1 [file 12864_2024_10072_MOESM1_ESM.docx]

**Supplemental Table 1: Resources used in the study**

| **Reagent or Resourse** | **Source** | **Identifier** | **Dilutions** |
| --- | --- | --- | --- |
| **Antibodies** | | | |
| Rabbit polyclonal PLZF | Santa Cruz | Cat# sc-22839 | 1：500 |
| Goat anti human LIN28A | R&D | Cat# AF3757 | 1：500 |
| Goat poly-clonal anti-GFRA1 | R&D | Cat# RLM3319 | 1：200 |
| Rabbit polyclonal to TBR2 / Eomes | Abcam | Cat# ab23345 | 1：50 |
| Goat polyclonal to GFP (FITC) | Abcam | Cat# ab6662 | 1：200 |
| Rat anti-TRA98 | Abcam | Cat# ab82527 | 1：400 |
| Rabbit polyclonal to Ki67 | Abcam | Cat# ab15580 | 1：200 |
| Anti-SOX9 Antibody | Sigma | Cat# ABE571 | 1：200 |
| Rabbit polyclonal to FPRL1/RFP | Abcam | Cat# ab63022 | 1：200 |
| c-Kit Rabbit mAb | CST | Cat# 3074S | 1：200 |
| Rabbit polyclonal H4k20me3 | Ruiying | Cat# RLM3279 | 1：400 |
| Rb (phospho Thr826) Polyclonal Antibody | Ruiying | Cat# RLP0556 | 1：400 |
| Rb (phospho Ser608) Polyclonal Antibody | Ruiying | Cat# RLP0774 | 1：400 |
| Thy1 Microbeads,mouse | Miltenyi Biotec | Cat#130-049-101 | 1：10 |
| Donkey anti Rabbit 488 | Abcam | Cat# ab150073 | 1：1000 |
| Donkey anti Mouse 594 | ProteinTech | Cat# SA00013-7 | 1：1000 |
| Donkey anti Rabbit 555 | Abcam | Cat# ab150074 | 1：1000 |
| HRP* Goat anti Rabbit | Ruiying | Cat# RS0002 | 1：500 |
|  | | | |
| **Primers for Genotyping** |  |  | |
| **Gene name** | **Primer Sequence** | **Product** | |
| Genotype Tbr2-eGFP A | ccgtctgcgattcgctaaa | Mutant :300bp  Wildtype :324bp | |
| Genotype Tbr2-eGFP B | tagcggctgaagcactgca |  |  |
| Genotype Tbr2-eGFP C | ctaggccacagaattgaaagatct |  |  |
| Genotype Tbr2-eGFP D | gtaggtggaaattctagcatcatcc |  |  |
| Genotype vasa-F | cacgtgcagccgtttaagccgcgt | Mutant :240bp  Wildtype :324bp | |
| Genotype vasa-R | tgcccattctaaacaacaccctgaa |  |  |
| Genotype vasa-CF | ctaggccacagaattgaaagatct |  |  |
| Genotype vasa-CR | gtaggtggaaattctagcatcatcc |  |  |
| Genotype Tbr2-F | agatggaaatttgggaatgaa | Mutant:731 bp  Wildtype: 621 bp | |
| Genotype Tbr2-R | ggctactacggcctgaaact |  |  |
| Genotype Tbr2-OE-A | catcaagctgatccggaacc | Mutant:307  Mutant:466 | |
| Genotype Tbr2-OE-B | cttcttggacgctttgtctaagtcc |  |  |
| Genotype Tbr2-OE-C | cttatcagaggaagatggcagct |  |  |
| Genotype Tbr2-OE-D | aagcagcgtatccacatagc |  |  |
| Genotype Lin28_YFP_F | ccccagttctcagggaaagcc | Knock in:236bp  Wildtype: 184bp | |
| GenotypeLin28_YFP_WTR | ccacccttacccccactttct |  |  |
| Genotype Lin28_YFP_TR | tgaacttgtggccgtttacgt |  |  |
| Genotype RG-TF | cccatggtcttcttctgcat | Mutant:220 bp  Wildtype: 324 bp | |
| Genotype RG-TR | aaggtgtacgtgaagcaccc |  |  |
| Genotype RG-CF | ctaggccacagaattgaaagatct |  |  |
| Genotype RG-CR | gtaggtggaaattctagcatcatcc |  |  |
|  | | | |
| **Primers for RT-PCR** | | | |
| **Gene name** | **Primer Sequence** | **Product** | |
| Tbr2 | ccgggacaactacgattcca | 100 bp | |
|  | acctccagggacaatctgatg |  |  |
| Rps2 | accaaatggcggatgacg | 238bp | |
|  | acgcacccaggaagaaat |  |  |
| Gapdh | aggtcggtgtgaacggatttg | 129 bp | |
|  | tgtagaccatgtagttgaggtca |  |  |
|  | | | |
| **Chemicals** | | | |
| DNase I | Sigma | Cat#D4527 | |
| Collagenase Type IV | Sigma | Cat# C2674 | |
| trypsin–EDTA solution | Gibco | Cat# R001100 | |
| Albumin from bovine serum | Sigma | Cat# A6003 | |
| Percoll | Sigma | Cat#P7828 | |
| EdU | Ribobio | Cat#C00003 | |
| Cell-Light EdU Apollo567 In Vitro Kit(100T ) | Ribobio | Cat#C10310-1 | |
| Busulfan | Sigma | Cat# B2635 | |
| **Experimental models: Strains** |  |  | |
| C57/B6J-129S1 background wild type |  |  | |
| mT/mG mice | The Jackson Laboratory | (Stock No. 007676) | |
| *Ddx4*-Cre mice | The Jackson Laboratory | (Stock No.018980) | |
| *Eomes*-eGFP mice | Beijing Biocytogen |  | |
