# Supplementary material for: Single-cell transcriptome analyses reveal critical regulators of spermatogonial stem cell fate transitions
Source: BMC Genomics. 2024 Feb 3;25:138. doi: 10.1186/s12864-024-10072-0 (PMC10837949; doi:10.1186/s12864-024-10072-0)
Supplement: Supplementary file 2 — Supplementary Material 2 [file 12864_2024_10072_MOESM2_ESM.docx]

**Supplemental Materials and Methods**

1. **Generation of Eomes conditional express line**

Mouse TG-ZQ-003 gene spans about 7.9kb on chromosome 9 forward strand (Eomes) was cloned and loxP sites were inserted at both ends of the transcription termination element. We used the Tol2 transposon system to stably allow large scale DNA inserts high frequency of transgene. The fragment CAG Pr-lox2-WPRE-pA from mROSA-KI-12(digested with PacI and MluI) was cloned into Tol2-2G-5`HS4-MC vector (digested with PacIand AscI) to form Tol2-CAG Pr-lox2-WPRE-pA vector. And then the fragment mTG-ZQ-003-CDS (synthesis) was ligated into Tol2-CAG Pr-lox2-WPRE-pA vector (digested with AscI and NheI) to form mTG-ZQ-003 transgene vector. The zygote microinjection was established through pronuclear. After genotyping and sequencing, the resulting positive mice were bred with 129S1 mice and their germline offspring were bred to establish stable lines. Eomes conditional overexpression (EomesOE) mice were maintained on a 129S1; FVB background. EomesOE females were mated with *Ddx4-cre* males to generate *Ddx4-cre; Eomes-cOE* males (expressing Eomes in the germline, designated as Eomes-cOE) and control littermates.

1. **Recipients for SSC transplantation**

F1 hybrids between C57BL/6J and 129S1/SvlmJ (Jackson Laboratories, stock no. 002448) were used as recipients for SSC transplantation (Yang et al. 2013). Intraperitoneal injection of busulfan (Cat. No. B2635, Sigma Chemical Co., St. Louis, MO) was performed to eliminate endogenous germ cells. Briefly, busulfan was dissolved in dimethyl sulfoxide (DMSO; Cat. No. D8418, Sigma Chemical Co.) at a concentration of 20mg/mL. Just before the injection, an equal volume of heated (37°C) sterile distilled water was added to reach a final concentration of 10.0 mg/mL. The final dose of busulfan was 44 mg/Kg of body weight.

1. **Lin28^+^ and Eomes^+^ spermatogonia isolation by FACS**

Testis from adult male mice were digested in 5ml trypsin enzyme for 5 min at 37°C, added 1 ml 1mg/ml DNase I, blow by pipette, and repeated this operation until testis were digested to be single cells. Adherent cells were filtered by 40 um cell strainers. Single cell suspension was pelleted by centrifugation at 400 g for 5 min, and resuspended in 5 ml DPBS-S. 5 ml cell suspension were added on 2 ml 30% precoll (Sigma, USA) in DPBS-S, and centrifugated at 1800 rpm for 8 min to wipe off apart of Sertoli cells and interstitial cells. DPBS-S washed cells for three times. Cells were added 1mL cell culture medium (90% dulbecco’s modified eagle medium (DMEM; Invitrogen), 10% FBS,1% penicillin-streptomycin). Yfp or Gfp^+^ cells were sorted by a Flow cytometer (BECKMAN COULTER, USA).

1. **RNA velocity analyses**

The RNA velocity was conducted with the velocyto R-package (version 0.17.17), according to the previous reports (La Manno et al. 2018), we used the spliced and unspliced transcript reads to calculate the RNA velocity. The spliced and unspliced reads were processed by R script velocity. R with the Cell Ranger output. All the Eomes positive spermatogonia were analyzed (n=1148). Velocity fields were projected onto the t-SNE plot generated by Seurat. Parameter n sight was 200 which determines the projection size of the velocity.

1. **qRT-PCR**

RNA was isolated using TRIzol reagent (Thermo Fisher Scientific, Germany) and purified using cDNA Synthesis SuperMix for qPCR kit (Novogene, China) according to the manufacturer’s instructions. qPCR was performed using SYBR Premix Ex Taq II (Novogene, China) and A ViiA7 Real Time PCR System (Applied Biosystems, USA) was used to quantify the relative abundance of Specific transcripts. Primer sequences are included in Supplemental Table 1. Differences between conditions were calculated using the ΔΔCt method.

**Reference**

La Manno G, Soldatov R, Zeisel A, Braun E, Hochgerner H, Petukhov V, Lidschreiber K, Kastriti ME, Lönnerberg P, Furlan A et al. 2018. RNA velocity of single cells. *Nature* **560**: 494-498.

Yang QE, Kim D, Kaucher A, Oatley MJ, Oatley JM. 2013. CXCL12-CXCR4 signaling is required for the maintenance of mouse spermatogonial stem cells. *J Cell Sci* **126**: 1009-1020.
