# Supplementary material for: Single-cell transcriptome analyses reveal critical regulators of spermatogonial stem cell fate transitions
Source: BMC Genomics. 2024 Feb 3;25:138. doi: 10.1186/s12864-024-10072-0 (PMC10837949; doi:10.1186/s12864-024-10072-0)
Supplement: Supplementary file 9 — Supplementary Material 9 [file 12864_2024_10072_MOESM9_ESM.docx]

**Supplemental Figure 1**


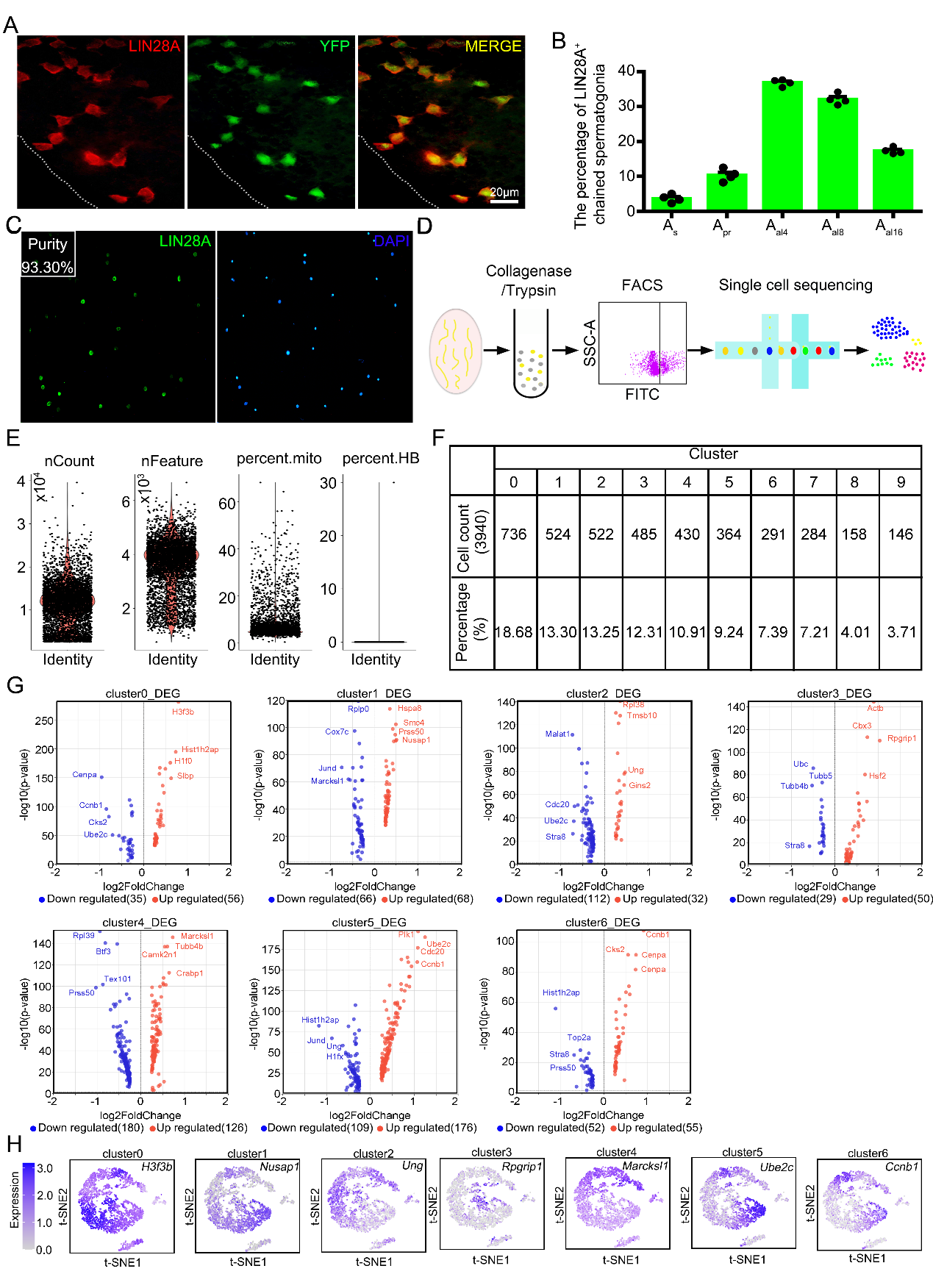


**Figure S1.** **Single-cell RNA-Seq of** **Lin28-YFP^+^ spermatogonia isolated from the testes of adult mice.**

**(A)** Whole-mount immunostaining of LIN28A and YFP expression in seminiferous tubules of testes from Lin28-YFP knock-in mice at PD60. Scale bar=20 μm, n=3. **(B)** The proportion of A_s_, A_pr_, A_al-4_, A_al-8_, and A_al-16_ spermatogonial clones in seminiferous tubules of testes from 2-month-old mice and at least 1500 LIN28A^+^ spermatogonia were counted for each sample. n=4. **(C)** Immunostaining for LIN28A in FACS-sorted YFP cells from the Lin28-YFP mouse line. **(D)** Schematic diagram of single-cell RNA-seq of Lin28-YFP single cells from adult mice. **(E)** Count number, gene number, mitochondrial gene ratio, and red blood cell gene ratio of each cell in scRNA-seq. **(F)** The number and proportion of cells in each cluster in the t-SNE plot of Lin28-YFP^+^ cells after stringent quality control. **(G)** Volcano plot of DEGs in clusters 0-6 (adjusted p value <0.05; fold change >2). Red indicates upregulated genes, and blue indicates downregulated genes. **(H)** Expression patterns of representative marker gene of clusters 0-6 projected on the t-SNE plots.

**Supplemental Figure 2**


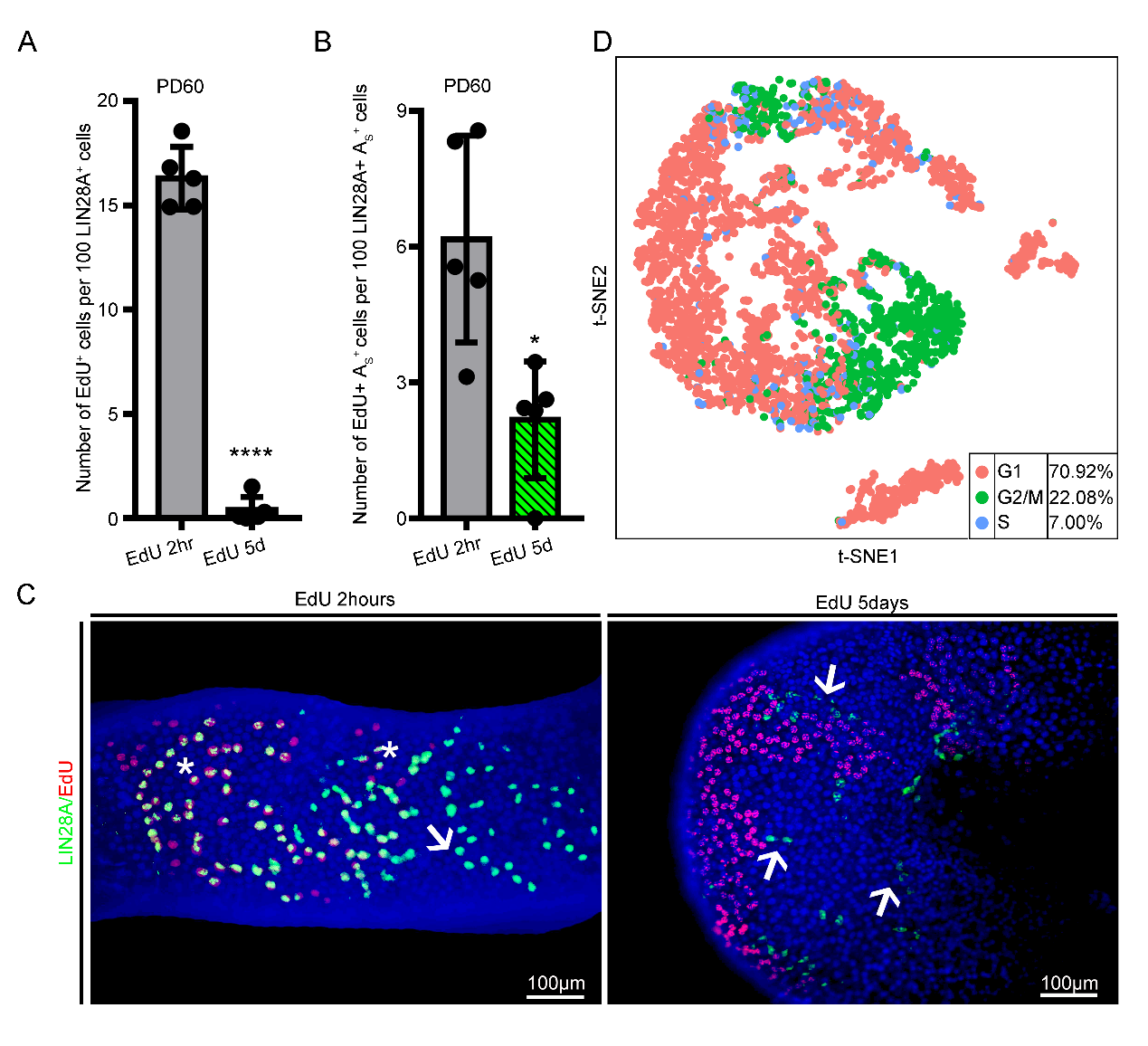


**Figure S2. Lin28-YFP single cells contained a quiescent subpopulation.**

**(A)** Quantifications of EdU^+^ spermatogonia in Lin28^+^ cells of testes collected 2 h and 5 days after EdU injection from 60-day-old mice. At least 1500 Lin28^+^ cells were counted for each time point. n = 5. ****p value < 0.0001. **(B)** Quantifications of EdU^+^ spermatogonia in A_s_ spermatogonia of Lin28^+^ cells of testes collected by EdU injection 2 h and 5 days from 60-day-old mice. At least 1500 LIN28^+^ cells were counted for each time point. n = 5. *p value < 0.05. **(C)** Whole-mount immunostaining of EdU and LIN28A in seminiferous tubules 2 h and 5 days after EdU injection. Scale bars = 100 μm, n=5. **(D)** Cell cycle analysis of Lin28-YFP single cells was performed using Seurat, reputation= t-SNE. G1, S and G2/M phases are indicated in the corresponding colors.

**Supplemental Figure 3**


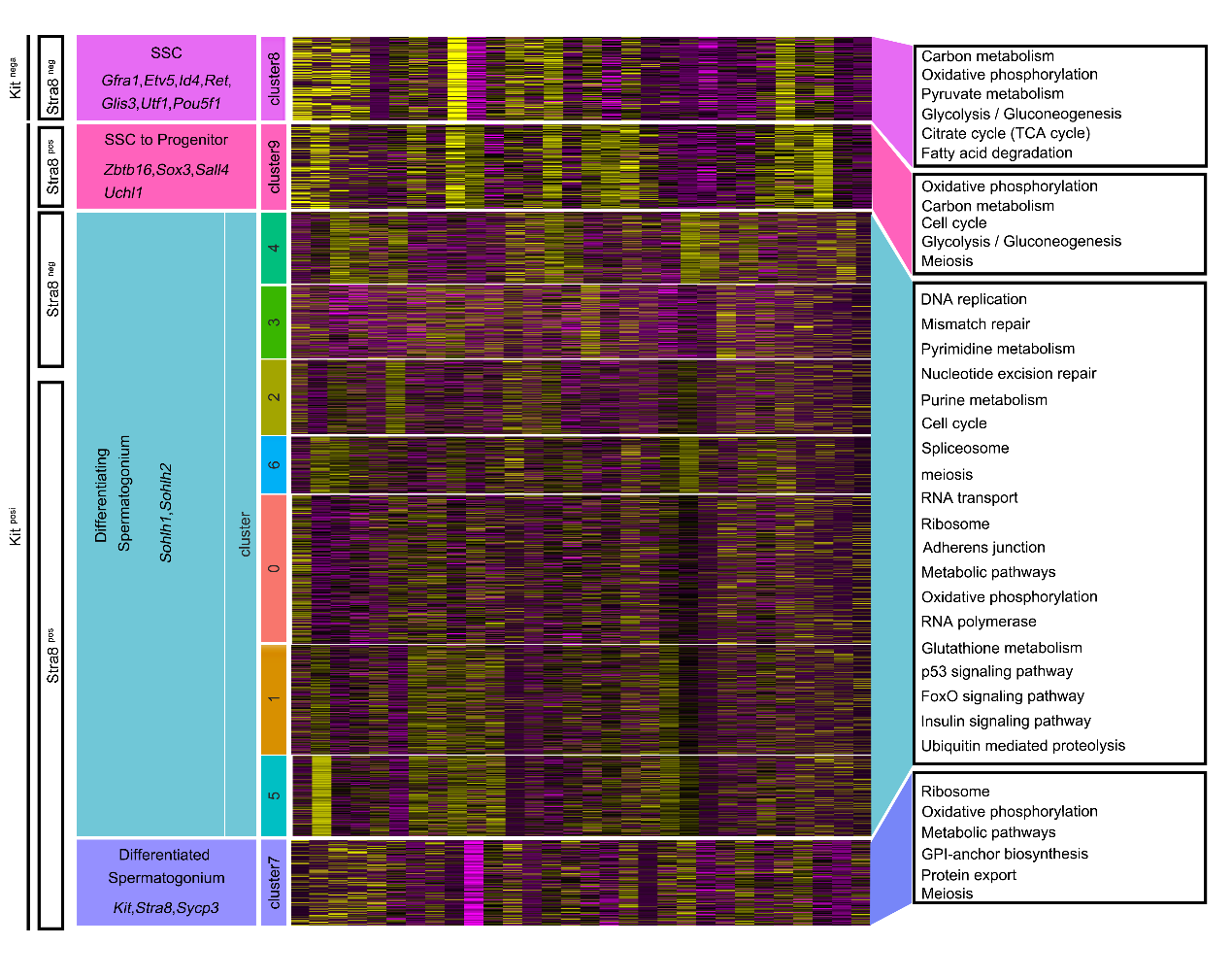


**Figure S3. Heatmap of cluster-specific genes in** **Lin28-YFP single cells.**

Lin28-YFP single cells were mainly divided into different states according to the t-SNE gene expression profile. Cluster 8 was putative SSCs, cluster 9 was progenitors, and cluster 7 was differentiating spermatogonia. Top 10 enriched KEGG terms for each temporal cell type cluster (right).

**Supplemental Figure 4**

**
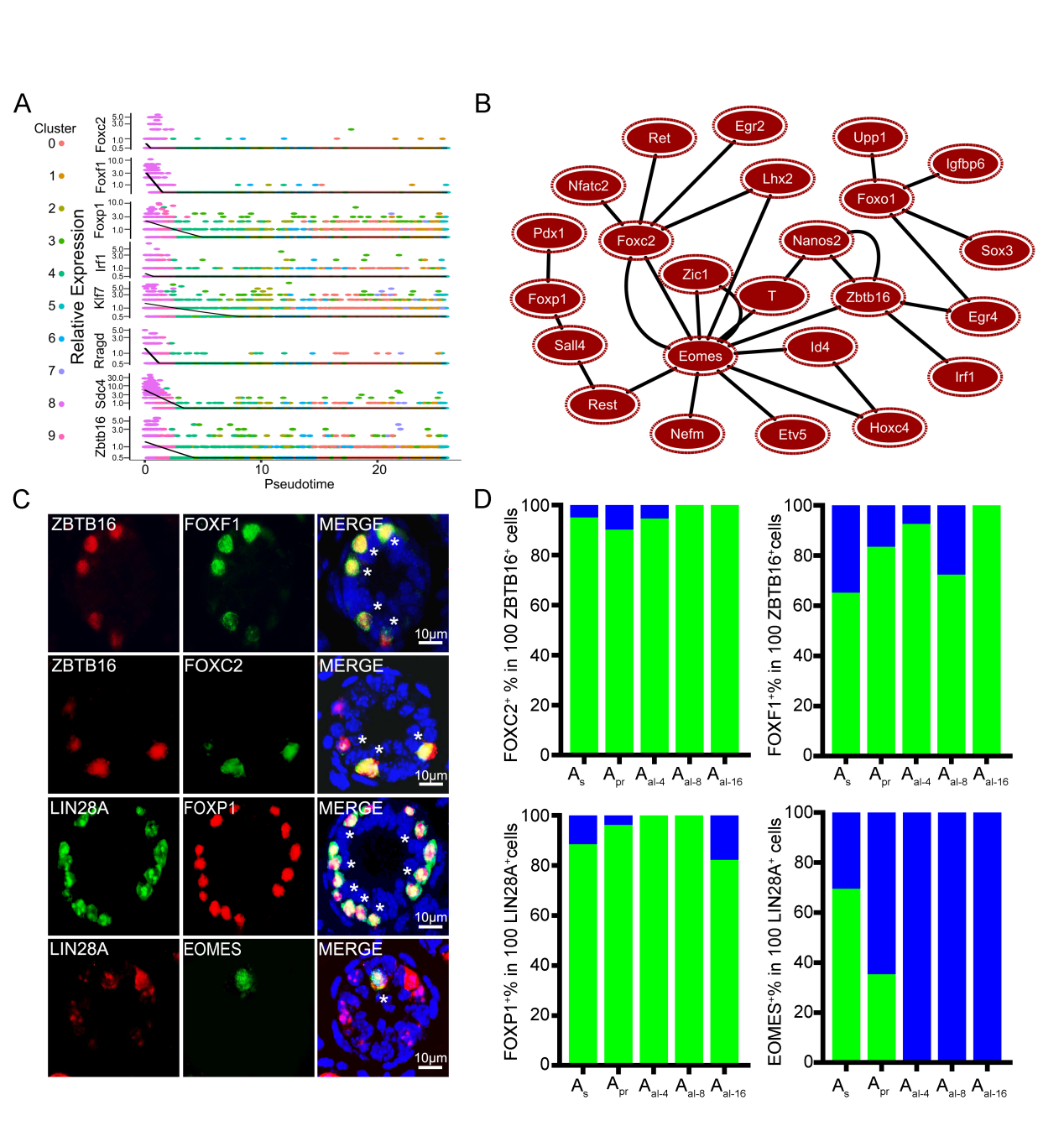
**

**Figure S4.** **Undifferentiated spermatogonial-specific transcription regulators expressed in C8.**

1. Differential expression patterns of new genes from C8 of genes along developmental pseudotime trajectory. **(B)** Undirected regulon network generated from strongly correlated scores by transcription factor regulons prediction in C8. **(C)** Immunofluorescence of ZBTB16 and LIN28A (undifferentiated spermatogonia) costained with FOXF1, FOXC2, FOXP1 and EOMES in 6-day-old mouse testicular paraffin sections. Asterisks indicate colocalization. Scale bars=10 μm, n=3. **(D)** Statistical results of immunofluorescent colocation of FOXF1, FOXC2, FOXP1 and EOMES in A_s_, A_pr_ and A_al_ spermatogonia of adult mice. At least 1000 LIN28A^+^ /ZBTB16^+^ cells were counted for each sample. n = 3.

**Supplemental Figure 5**

**
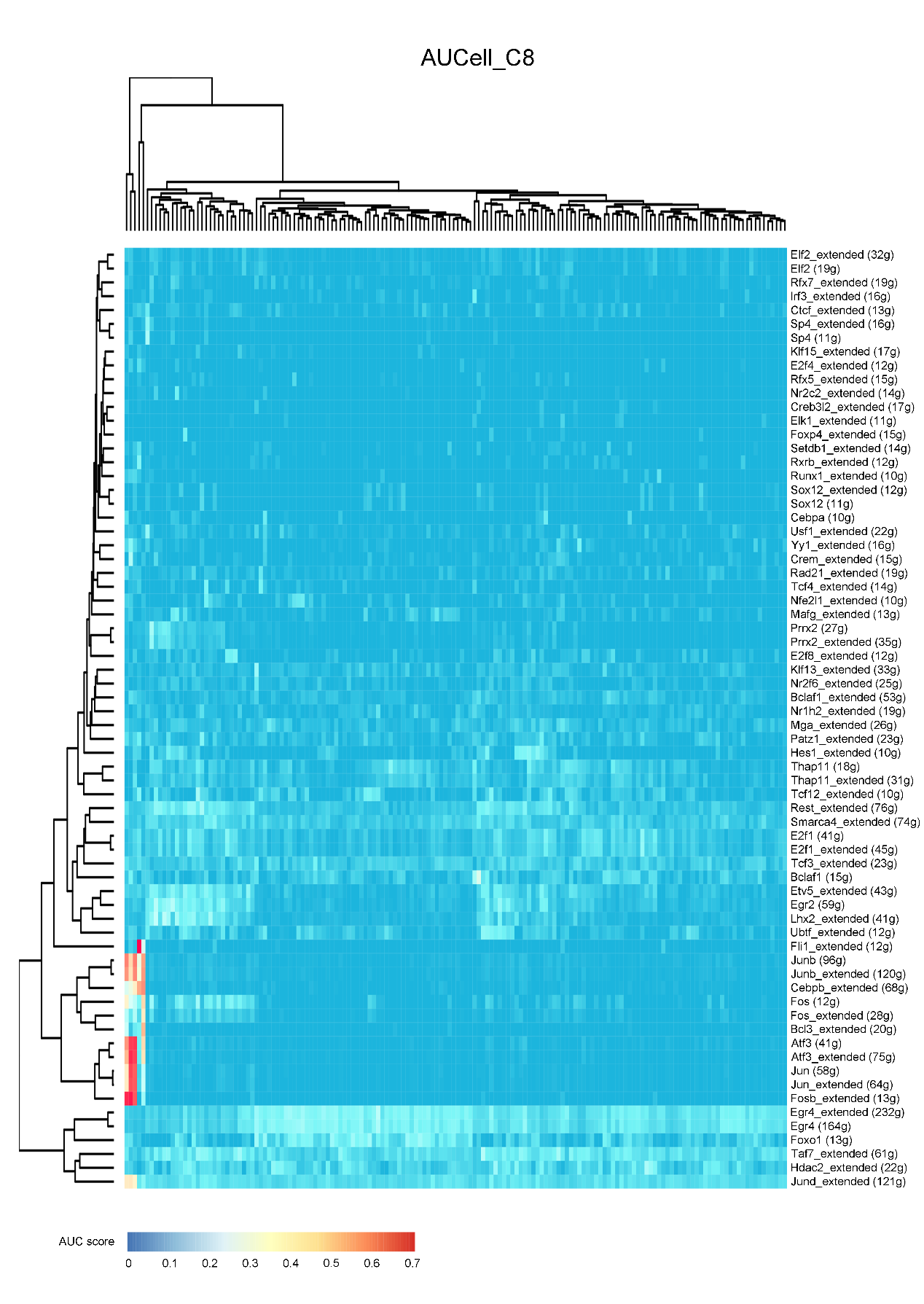
**

**Figure S5. Regulon activity heatmap of C8.** Heatmap of regulon activity scores. Hierarchically clustered regulons are listed on the left and top sides.

**Supplemental Figure 6**


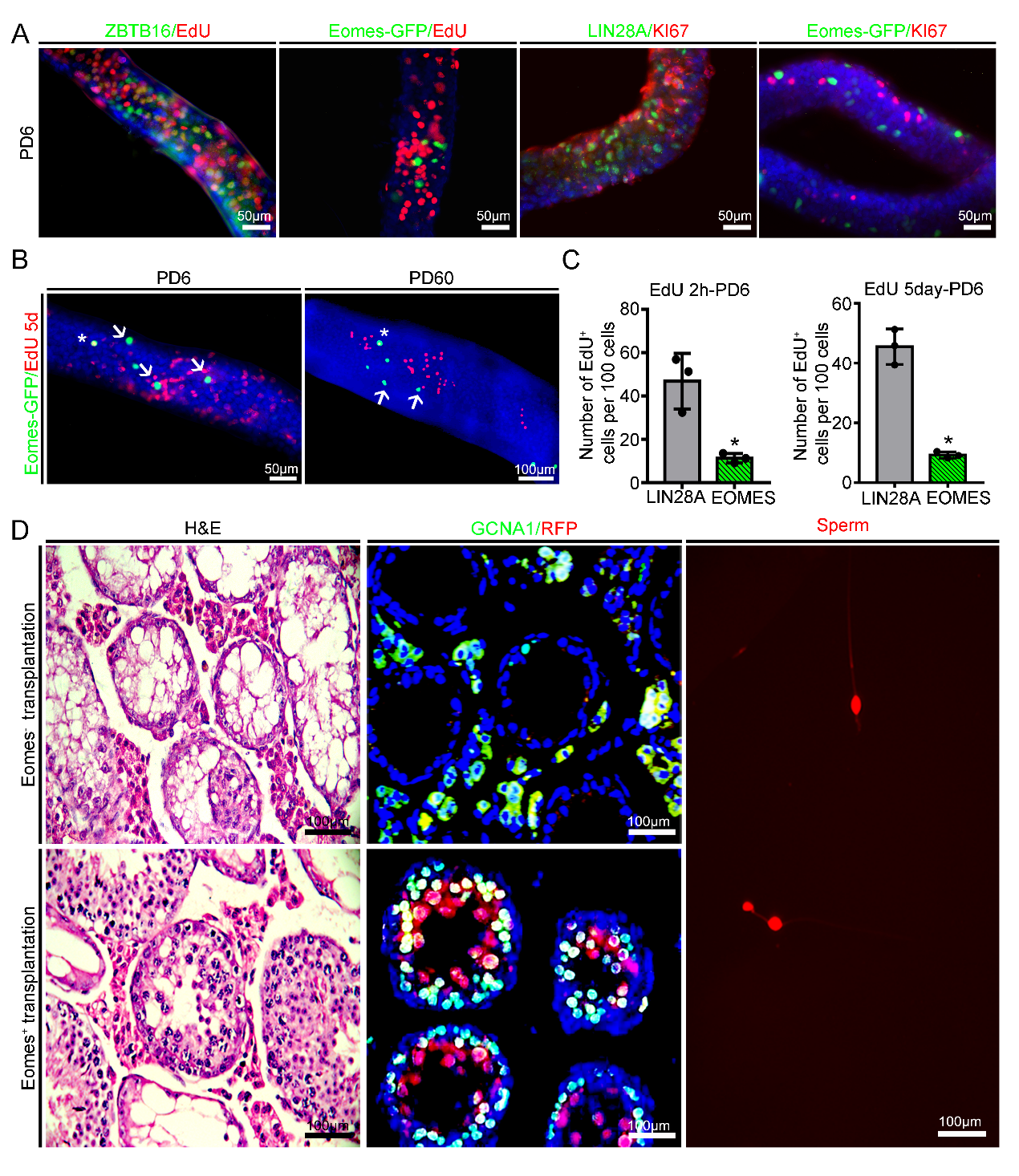


**Figure S6. Eomes^+^ spermatogonia act as SSCs after transplantation.**

**(A)** Whole-mount immunostaining of EdU & LIN28A, KI67 & LIN28A, EdU & EOMES, and KI67 & EOMES in seminiferous tubules at 2 h after EdU injection at PD6. Scale bars=50 μm, n=3. **(B)** Whole-mount immunostaining of EdU & EOMES in seminiferous tubules 5 days after EdU injection at PD6 and PD60. Scale bars=50 μm/100 μm, n=3. **(C)** Statistical results of EdU & EOMES in seminiferous tubules after 5 days of EdU injection. At least 1000 LIN28A^+^ /EOMES^+^ cells were counted for each sample, ns means no significance. **(D)** Transplantation of Eomes-GFP-positive and Eomes-GFP-negative cells and after 2 months of recipient testes collection, (left) morphology of testicular tissue observed by H&E, (middle) germ cell staining to determine the origin of SSC and (right) red spermatozoa derived from Eomes-positive donor cells.

**Supplemental Figure7**

**
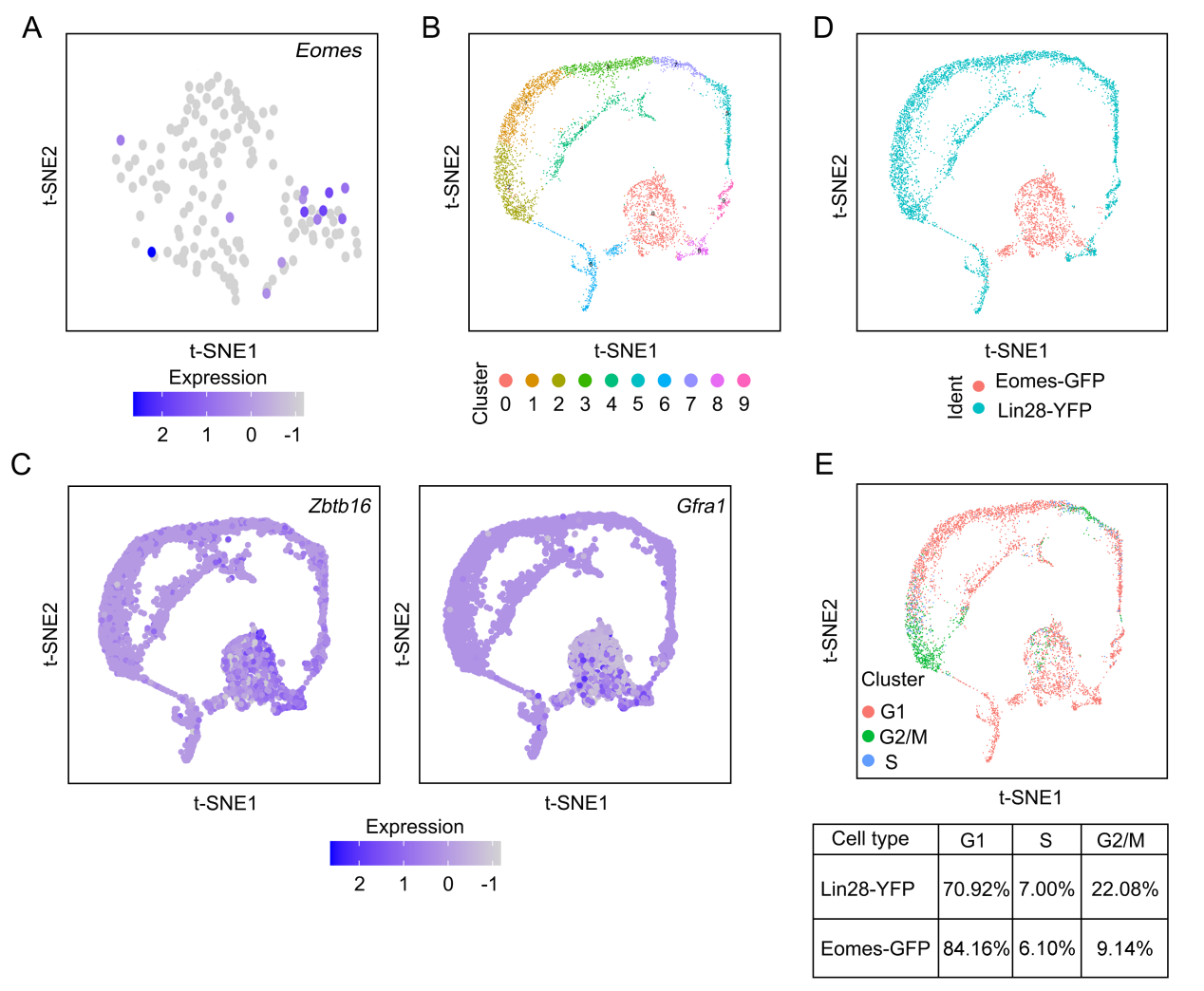
**

**Figure S7. Eomes^+^ spermatogonia contained more quiescent cells than Lin28-YFP single cells.** **(A)** Gene expression patterns of *Eomes* projected on the t-SNE plots in C8 of Lin28-YFP single cells. **(B)** t-SNE plot of integrated Lin28-YFP^+^ spermatogonia and Eomes-GFP^+^ spermatogonia. Each dot represents a single cell, and cell clusters are distinguished by colors. **(C)** Gene expression patterns of selected marker genes (*Zbtb16* and *Gfra1*)projected on the t-SNE plots. **(D)** t-SNE plot of the integrated Lin28-YFP^+^ spermatogonia and Eomes-GFP^+^ spermatogonia single-cell data. Each point represents a cell. Points were colored according to cell type. **(E)** Cell cycle analysis of integrated Lin28-YFP^+^ spermatogonia and Eomes-GFP^+^ spermatogonia. G1, S and G2/M phases are indicated in the corresponding colors.The proportion of G1, S and G2/M phases cells in different cell types were shown in the table.

**Supplemental Figure8**

**
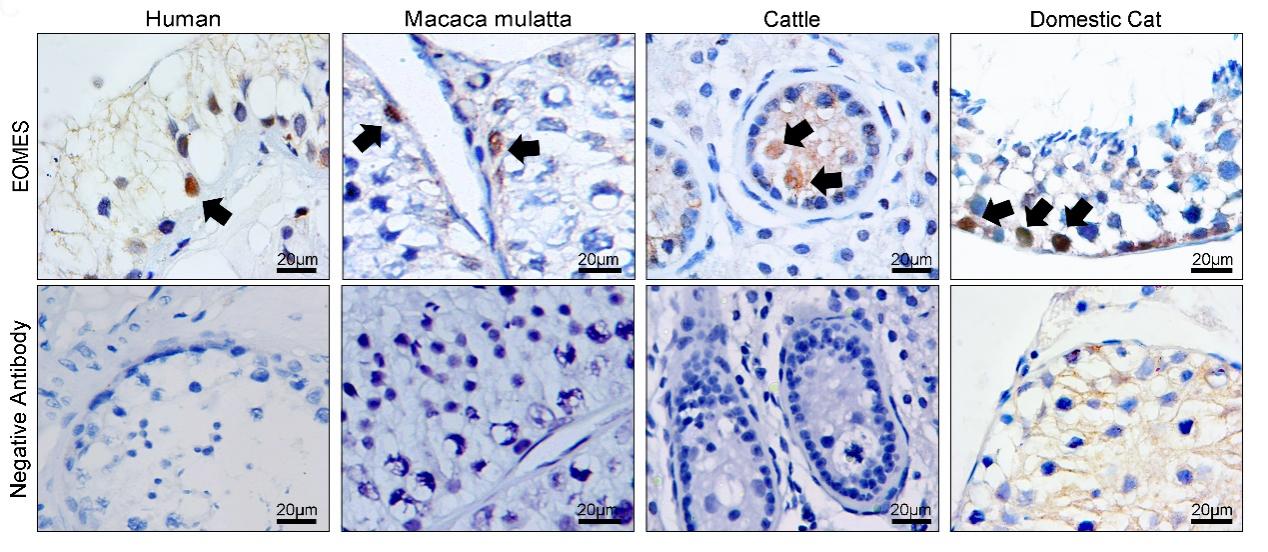
**

**Figure S8. Distribution of Eomes^+^ spermatogonia in the testes of different species.** Eomes+ cells were detected in the testes of humans, monkeys, cattle, and cats. Arrowheads show localization of signal to Eomes^+^ spermatogonia. Scale bars=20 μm.

**Supplemental Figure 9**


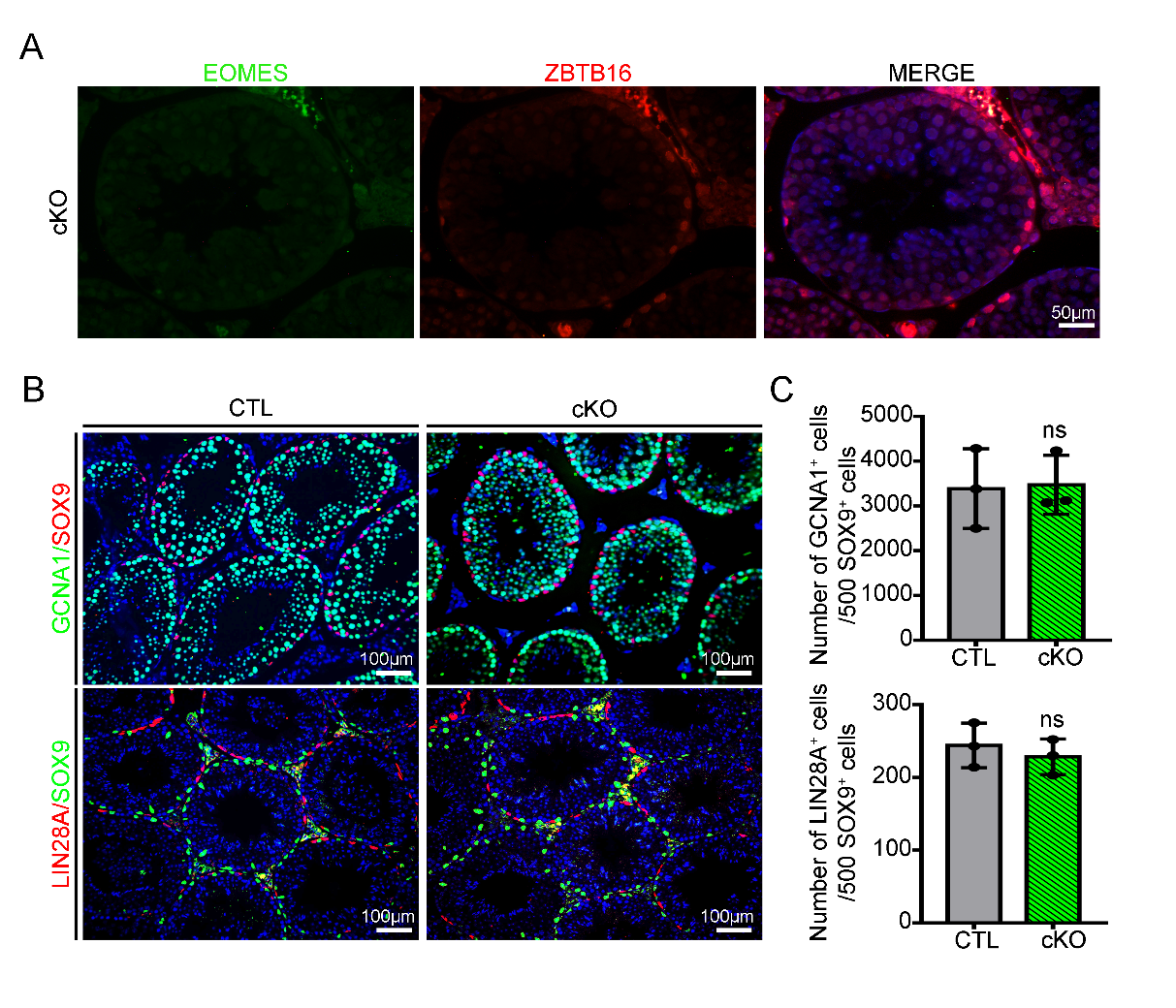


**Figure S9. *Eomes* deletion in the germline did not affect steady-state spermatogenesis.**

**(A)** Immunofluorescent costaining for ZBTB16 with EOMES in cross-sections of testes from adult Eomes-cKO mice. Scale bar=100 μm, n=3. **(B)** Immunofluorescent costaining for GCNA1 or LIN28A with SOX9 in cross-sections of testes from adult control and Eomes-cKO mice. Scale bars=100 μm, n=3. **(C)** The numbers of germ cells (GCNA1^+^) or undifferentiated spermatogonia (LIN28A^+^) per 500 Sertoli cells (SOX9^+^). At least 500 SOX9^+^ cells were counted for each sample, ns means no significance, n=3.

**Supplemental Figure10**


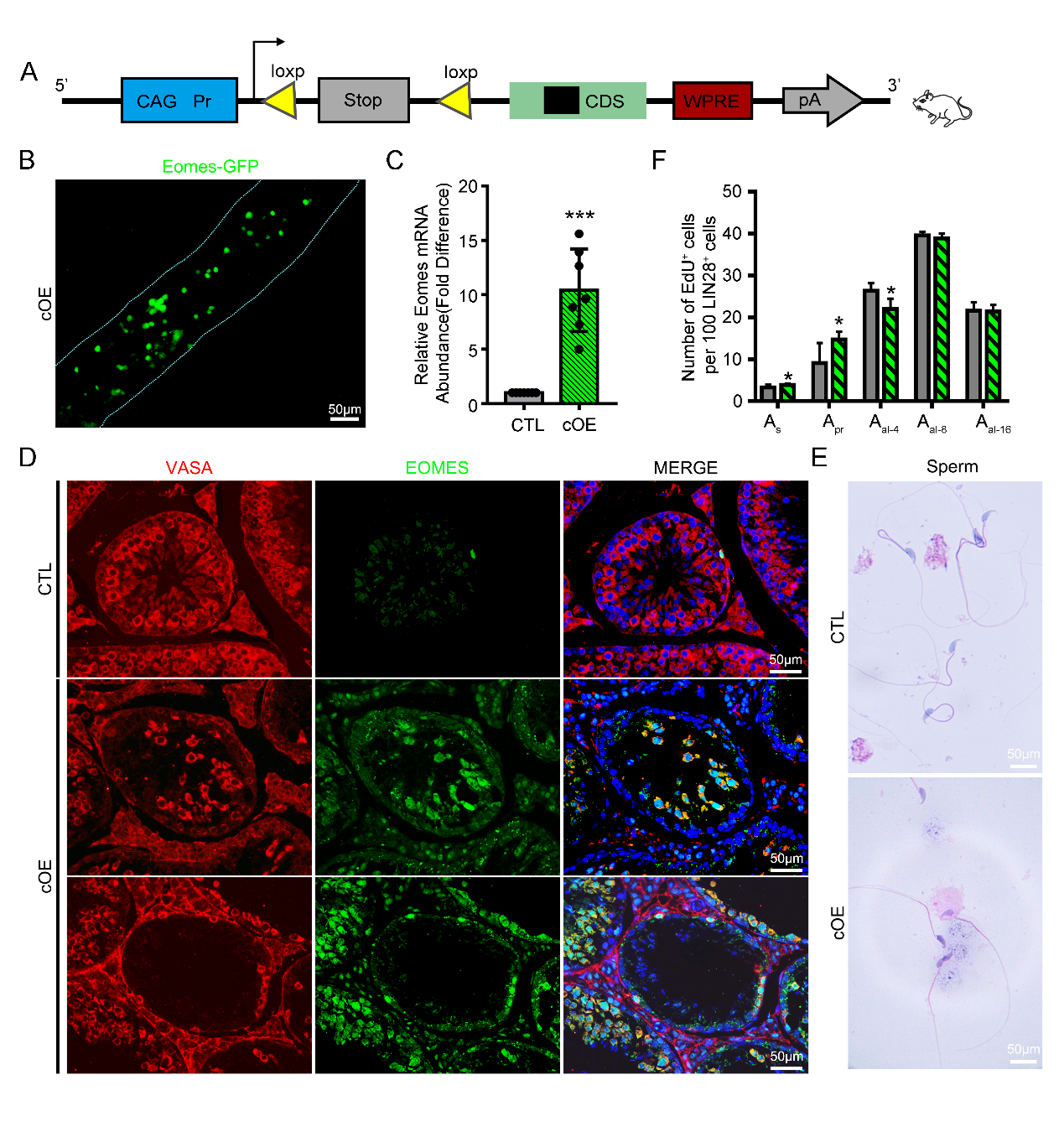


**Figure S10.** **Effects of forced *Eomes* expression on spermatogenesis in mice.**

**(A)** Schematic diagram of the transgene used for generating the conditional *Eomes* overexpression model. Cre-mediated recombination of the LoxP sites removes the stop cassette and activates *Eomes* expression under the direction of the CAG promoter. **(B)** Whole-mount immunofluorescent staining for EOMES in seminiferous tubules of Eomes-cOE mice. Scale bar=50 μm, n=3. **(C)** Relative concentration of *Eomes* transcripts in the testes of control and Eomes-cOE mice. Data are mean ± s.e.m. for 7 independent experiments. ***p value <0.001. **(D)** Immunofluorescence staining for the germ cell markers VASA and EOMES in testicular cross-sections from Eomes-cOE mice. Scale bars=50 µm, n=3. **(E)** Hematoxylin and eosin (H&E)-stained sperm from control and Eomes-cOE mice at PD90. Scale bars=50 μm, n=3. **(F)** Quantifications of EdU^+^ cells in LIN28A^+^ spermatogonia using whole mount staining of Eomes-cOE testes. At least 1500 LIN28A^+^ cells were counted for 3 independent experiments. *p value < 0.05, n=3.

**Supplemental Figure 11**


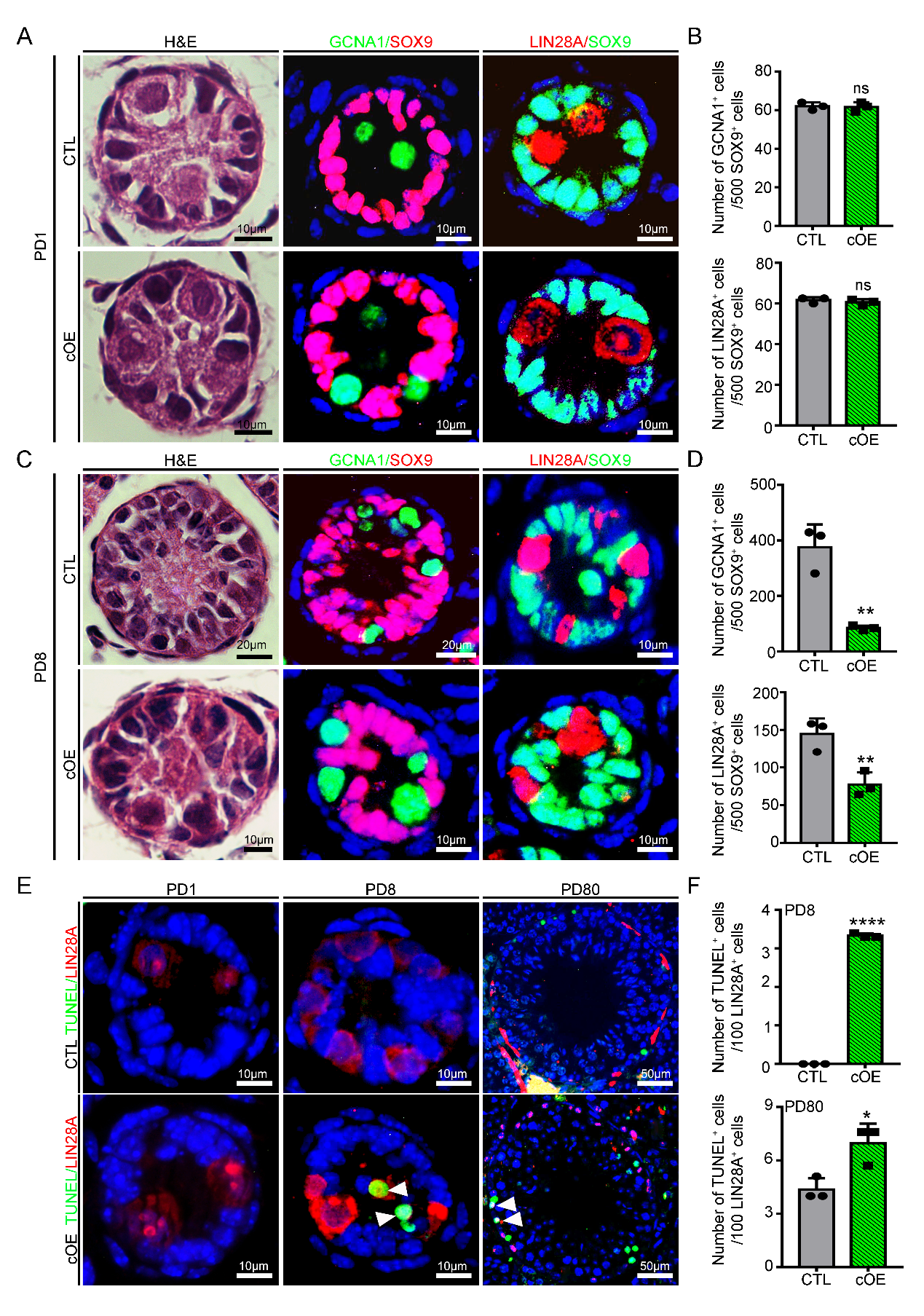


**Figure S11. Forced *Eomes* overexpression in the germline impaired spermatogenesis beginning at PD8.**

**(A)** Histological analysis and immunofluorescent costaining for GCNA1 or LIN28A with SOX9 in testicular cross-sections from testes of control and Eomes-cOE mice at PD0. Scale bars=10 µm, n=3. **(B)** Quantification of GCNA1^+^ cells and LIN28A^+^ cells per 500 SOX9^+^ cells supported at PD0. At least 500 SOX9^+^ cells were counted for each sample, ns means no significance, n=3. **(C)** Histological analysis and immunofluorescent costaining for GCNA1 or LIN28A with SOX9 in testicular cross-sections from testes of control and Eomes-cOE mice at PD8. Scale bars=10/20 µm, n=3. **(D)** Quantification of GCNA1^+^ cells and LIN28A^+^ cells per 500 SOX9^+^ cells at PD8. At least 500 SOX9^+^ cells were counted for each sample, n=3. **p value<0.01. **(E)** Immunofluorescent costaining for LIN28A and TUNEL (TdT-mediated dUTP nick-end labeling) in cross-sections of testes from control and Eomes-cOE mice at different developmental stages. Scale bars=10/50 µm, n=3. **(F)** Quantification of TUNEL^+^ cells per 100 LIN28A^+^ undifferentiated spermatogonia at different developmental stages. At least 500 SOX9^+^ cells were counted for 3 independent biological samples, *p value <0.05 and ****p value <0.0001.

**Supplemental Figure 12**

**
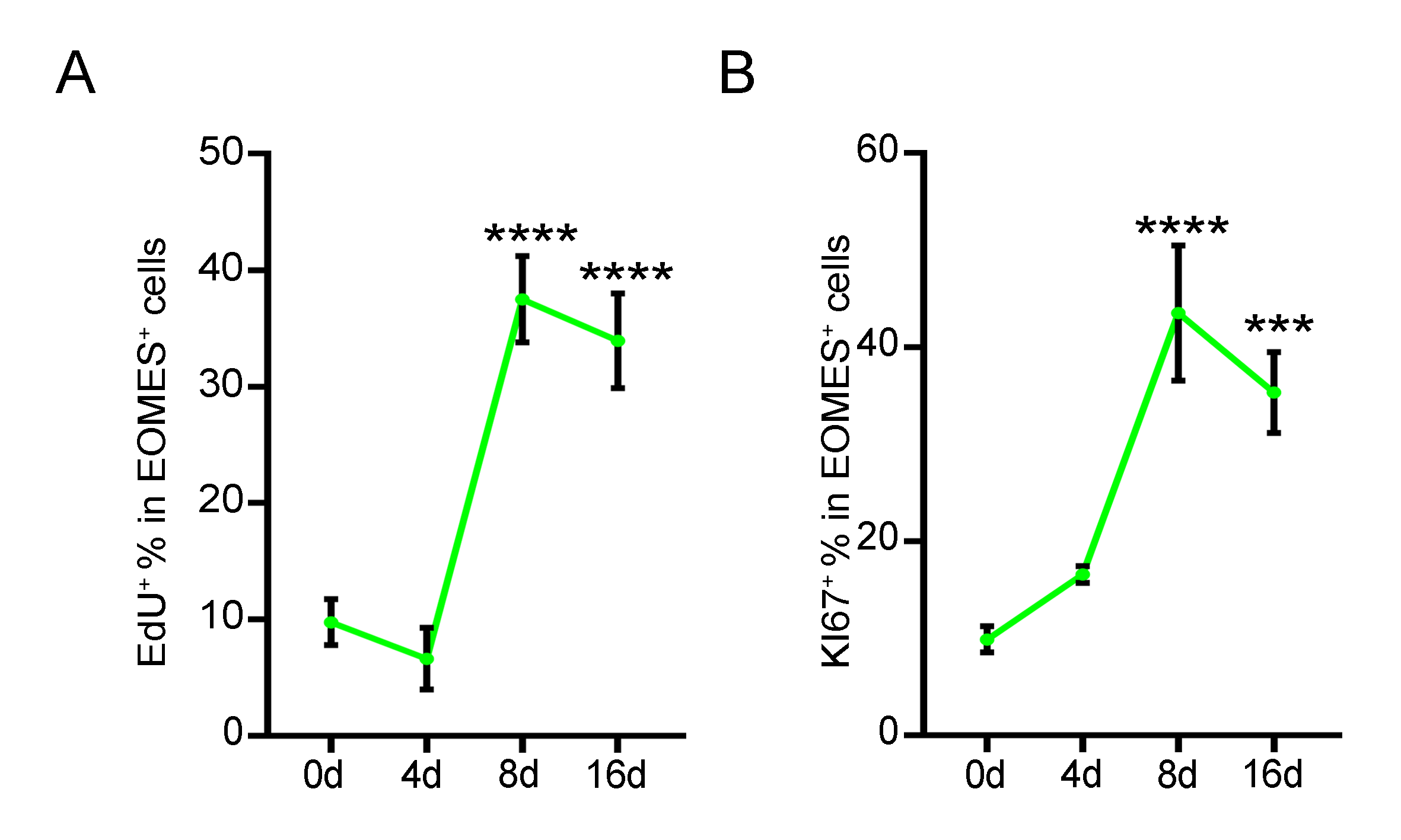
**

**Figure S12. The proliferation of Eomes^+^ spermatogonia after busulfan-induced testicular injury.**

**(A)** The ratios of EdU^+^ EOMES^+^ cells in testes from mice at different times were treated with busulfan (20 mg/kg body weight). Data are mean±s.e.m. for three independent experiments. At least 200 EOMES^+^ cells were counted for each sample. ****p value < 0.0001. n=3.**(B)** The ratios of KI67^+^EOMES^+^ cells in testes from mice at different times were treated with busulfan (20 mg/kg body weight). Data are mean±s.e.m. for three independent experiments. At least 200 EOMES^+^ cells were counted for each sample. ***p value<0.001 and ****p value < 0.0001. n=3.

**Supplemental Figure13**


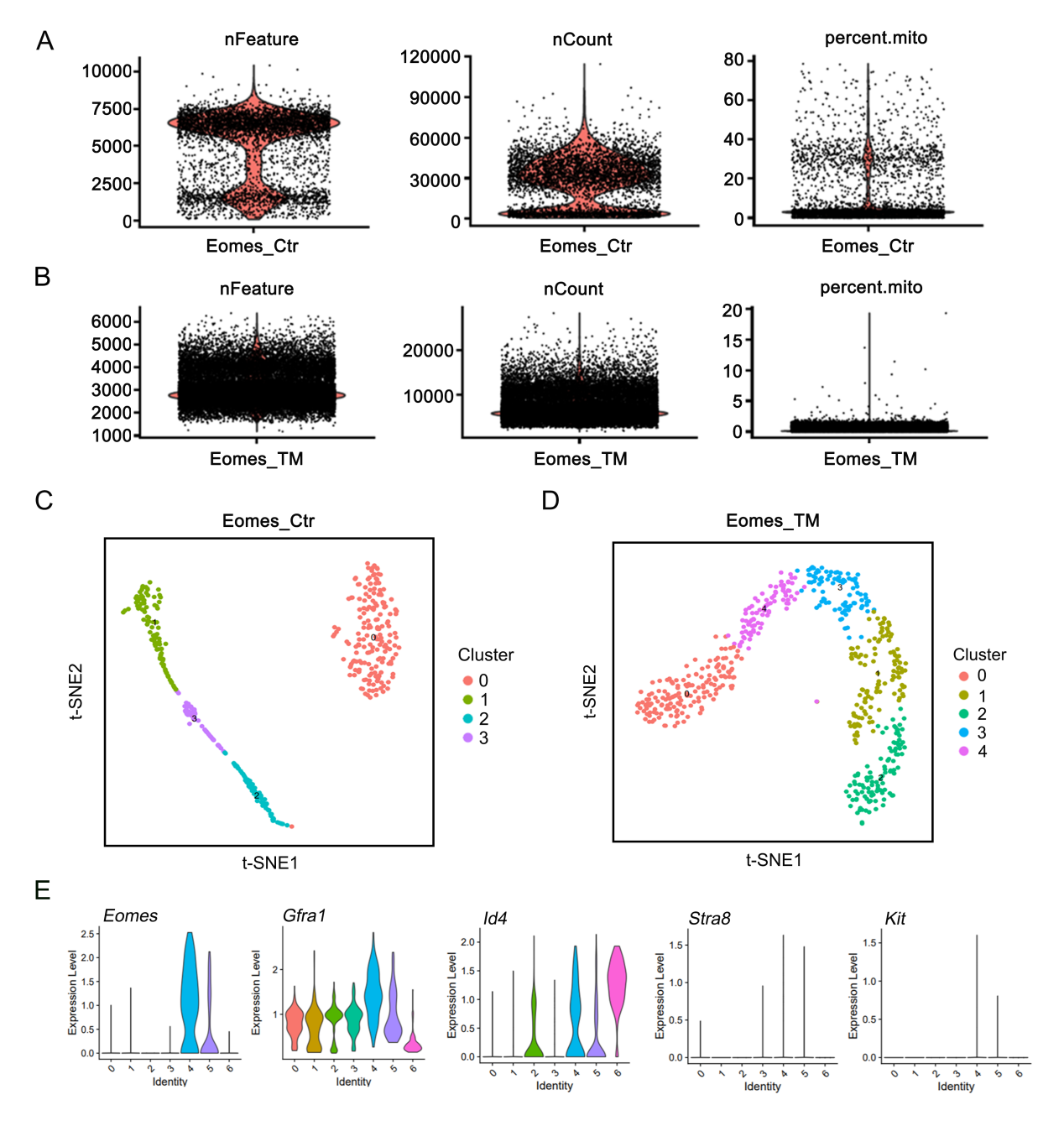


**Figure S13.Single-cell RNA-Seq of Eomes^+^ spermatogonia isolated from the testes of adult mice.**

**(A)** Count number, gene number, mitochondrial gene ratio, and red blood cell gene ratio of each cell in scRNA-seq of Eomes+ spermatogonia from control (Eomes-Ctr) . **(B)** Count number, gene number, mitochondrial gene ratio, and red blood cell gene ratio of each cell in scRNA-seq of Eomes+ spermatogonia from busulfan treatment day8 mice(Eomes-TM) **(C)** t-SNE plot of Eomes+ spermatogonia clusters from Eomes-Ctr defined by scRNA-seq analysis. Each dot represents a single cell, and clusters are marked by different colors. **(D)** t-SNE plot of Eomes+ spermatogonia clusters from Eomes-TM defined by scRNA-seq analysis. Each dot represents a single cell, and clusters are marked by different colors. **(E)** Violin plots of SSC, progenitor and differentiated spermatogonial markers in different clusters of integrated Eomes+ spermatogonia from Eomes-Ctr and Eomes-TM.
